# Modeling and simulation-assisted strategies for effective membrane-fouling mitigation during membrane bioreactor operation

**DOI:** 10.1016/j.heliyon.2024.e38953

**Published:** 2024-10-04

**Authors:** Maryam Homayoonfal, Zohre Hajhashemi, Maryam Hajheidari, Fateme Rezaei, Mohammad Saber Nadali

**Affiliations:** Department of Chemical Engineering, College of Engineering, University of Isfahan, P.O. Box 81746-73441, Isfahan, Iran

**Keywords:** Membrane bioreactor, The fouling development models, GPS-X simulation, In-series resistance model, Cake characteristics

## Abstract

This research principally aimed to present a suitable strategy for membrane-fouling mitigation in membrane-bioreactors (MBRs). The current strategies for membrane-fouling mitigation before initiating the process in many cases, are unmodifiable for a specific MBR system along the operations. Thus, membrane-fouling strategies during filtration should be applied. To select the best and most economical method for controlling fouling during the operations, the quality (site and mechanism) as well as quantity (thickness, mass, and porosity of the cake layer, and pore resistances) of fouling should be predicted. Accordingly, in this research, two powerful tools, i.e. modeling and simulation, have been used for predicting the quality and quantity of fouling, respectively. Through modeling, the best model describing the site and mechanism of fouling was chosen. Through simulation, the thickness, mass and porosity of the cake layer, along with resistance of cake and pores were calculated. In addition, the match between the results of modeling, simulation, and experimental results confirmed the accuracy of the performed predictions. Ultimately, to achieve the minimum membrane-fouling during filtration, based on the modeling results, the general solution of washing (physical or chemical), and based on the simulation results, its intensity (low, medium, and high) were proposed.

## Highlights

• The most efficient method for membrane fouling mitigation inside MBR was proposed.
• The quality of fouling (site and mechanism) was determined via modeling.
• The quantity of fouling (cake and pore resistances) was determined via simulation.
• Based on modeling, the best washing strategy (physical or chemical), was proposed.
• Based on simulation, the washing intensity (low, medium, and high), was proposed.

## Graphical abstract

**Figure:**
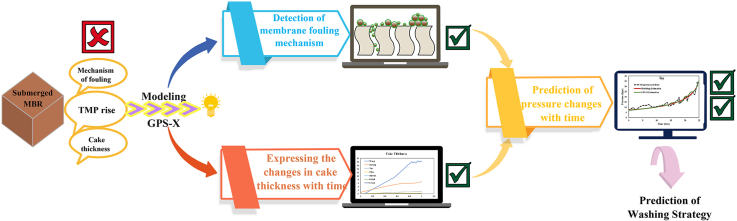

## 1 Introduction

Membrane bioreactor (MBR) is an advanced and hybrid technology of activated sludge and membrane filtration, producing a treated effluent with a higher quality compared to the conventional activated sludge process [1,2]. The advantages of this technology include small space occupation, low sludge production, high removal of biological oxygen demand (BOD) and chemical oxygen demand (COD), high biodegradation efficiency, and high quality of treated wastewater [3]. Nevertheless, membrane fouling (especially biological fouling) is among the most important challenges of MBR, causing reduced separation efficiency, dramatic decline of the permeation flux, or rapid increase in transmembrane pressure (TMP) as well as higher operational costs, all limiting the practicality of this technology [4].

The factors affecting membrane fouling include the properties of wastewater and biomass (carbon to nitrogen to phosphorus ratio (C/N/P), presence of coagulant and salts such as sodium chloride (NaCl), pH) [[5], [6], [7], [8]], membrane properties (surface hydrophilicity and size of pores) [[9], [10], [11], [12]], as well as operational conditions (temperature, solid retention time (SRT), and hydraulic retention time (HRT)) [13]. For example, control of pH within the neutral range [14], adding coagulants (alum) [15], elevation of C/N/P ratio [16], and reduction of NaCl concentration [17] could reduce TMP rise along the operation by up to 50, 52, 80, and 85 % respectively. Thus, it seems that controlling the ionic strength regarding the properties of wastewater and biomass is the most effective factor for reducing fouling. From among membrane properties, enhancement of hydrophilicity [18] and size of pores [19] contributed to lowering TMP growth along the operation by up to 75 and 84 % respectively, thereby clarifying the important role of pore size in controlling fouling. In the case of operational conditions, the elevation of SRT [20] and HRT [21], as well as the reduction of temperature [22], managed to diminish TMP growth by up to 85, 85, and 91 % respectively. Thus, it seems that temperature reduction would have a positive impact on the reduction of membrane fouling. Consequently, although increasing membrane pore size, decreasing temperature, and regulating ionic strength have been shown to reduce membrane fouling, given the absence of an instrument for precise control of ionic strength in the wastewater, the decline of separation with enlargement of pore size, as well as mortality of microorganisms with temperature reduction, the mentioned solutions have executive constraints.

Given the mentioned executive limitations in experimental dimensions, as well as the ability of methods that are based on modeling and simulation in predicting the quality of fouling (place of its incidence and mechanisms) and quantity of fouling (thickness, mass, and porosity of the cake layer, and pore resistances), numerous researchers have employed these two powerful tools for predicting membrane fouling and presenting optimal solutions for controlling it [[23], [24], [25]].

Kim, Sankararao [26] discovered that with prolongation of filtration time in a MBR, the fouling mechanism shifted from intermediate blocking to cake formation. Gao, Yang [27] found that increasing the ozone dose in pre-ozonation (from 0.5 mg O^3^/mg DOC to 1.5 mg O^3^/mg DOC) resulted in a thinner, denser cake layer and changed the fouling mechanism from cake to standard-intermediate. Bai, Wu [28] noted that during ozonation, increasing the ozone dose (from 0.05 mg O^3^/mg C to 2 mg O^3^/mg C) led to an increased share of fouling by particles with lower molecular weight (MW < 4000 Da) compared to higher molecular weight (MW > 4000 Da). Kirschner, Cheng [29] defined the threshold flux as the flux below which cake accumulation would be negligible (dominated by the intermediate pore blocking model) and above which it would increase (dominated by the combined intermediate pore blocking/cake filtration model), suggesting operational flux should be kept below this value. Jiang, Ngo [21] modeled experimental data and found that membrane fouling develops by two mechanisms of cake formation on the surface as well as intermediate blocking of pores. Cerón-Vivas, Kalboussi [30] through modeling and identifying the precise site of membrane fouling (cake formation on the surface and standard blocking of pores), corrected the process of membrane washing (replacing chemical washing with gas bubbling method). Sabia, Ferraris [31] through MBR modeling found that the history of the membrane (i.e. age, lifetime, etc.) has a greater effect on the fouling mechanism than the applied operating conditions (SRT and pre-denitrification). Also, the study by Xiong, Zuo [32] confirmed the lack of effect of the retention time of activated carbon inside the bioreactor on the type of fouling mechanism (cake formation and complete pore blocking) through modeling. Thus, modeling-based methods by spending less time and cost, in addition to boosting knowledge and predicting the qualitative properties of fouling (site and mechanism of fouling), can also present the optimal operational conditions for minimizing the fouling and determining the time of membrane cleaning [[33], [34], [35], [36]].

On the other hand, there are various advanced software applications for simulating water and wastewater systems including AQUASIM [37], BioWin [38,39], SIMBA [40], and GPS-X [41,42], which can predict optimal operational conditions by employing developed models [43]; thus, without any need for experimental equipment or special costs, they provide the results obtained from the operations as well as inaccessible results (thickness and porosity of the cake layer) with greater details and accuracy [36,44]. Among these, GPS-X stands out from the other software due to its high flexibility and ease of use. For instance, Al-Sayed et al. [45], aimed to reduce the effort and costs associated with conducting MBR operations on a full scale. They successfully simulated a laboratory system using GPS-X on a full scale and managed to predict MLSS/MLVSS with an impressive accuracy of 0.6 %. Similarly, Bis was able to forecast the levels of TP and PO_4_^3−^ in actual full-scale wastewater by simulating it with GPS-X [46]. Further, to control oxygen transfer efficiency, by simulating experimental data via GPS-X software, Sonawane and Murthy [47] were able to make the proper decision in selecting the type of aeration (fine bubbles instead of coarse bubbles). Also, the simulation conducted by Abdel-Kader [48] regarding the effect of the type of input wastewater on the quality of output effluent, led to the selection of a proper method for complementary treatment to improve the quality of the outlet effluent. Although in recent years GPS-X software has been able to offer acceptable results in the prediction of the performance of treatment processes including MBR such as MLSS, MLVSS, TP, COD, TKN [43], so far it has not been employed for predicting and controlling membrane fouling in MBR.

In the present research, modeling has been used for predicting the quality (site and mechanism) of fouling, while simulation via GPS-X software has been employed for predicting the quantity of fouling (thickness, mass, and porosity of cake layer, resistance of pores). For this purpose, in the first step, research on the use of MBR in wastewater treatment has been reviewed, from among which those dealing with submerged membrane and with a constant flux in MBRs which included essential information for the simulation (temperature, aeration rate, membrane flux, HRT, and SRT) were chosen for further investigation. In the second step, the mechanism of fouling was identified using well-known developed models, whereby the potential sites of fouling incidence (membrane surface and inside pores) were determined. In the third step, the performance of the MBR was simulated by GPS-X software. In this regard, first, the simulation results were confirmed against experimental results, and then through precise calculation of the cake and pore resistances, the modeling results were confirmed. Ultimately, precise information related to the quantity of fouling which is not easily measurable in the laboratory including the thickness, mass, and porosity of the cake layer was calculated. In the end, based on the obtained results, the possible solutions for reducing fouling were presented along with the type and intensity of their application along the operations.

## 2 Methodology

### 2.1 Data collection

Since MBR systems have diversity in the configuration (submerged and external) and performing operations (constant pressure or flux), in the simulation and modeling performed in the present research, only submerged one-stage MBR systems with a constant flux have been considered as the target of study because of lower fouling and thus lower costs [49,50]. In this regard, the examined studies (33 research) were categorized with three approaches of modifying the properties of the inlet wastewater and biomass, membrane, and operational conditions. Next, for each research, the data related to the MBR system including the physical properties of the bioreactor, inlet wastewater properties, and operational conditions of the bioreactor were extracted. The best data (relating to fouling extent) were chosen and used for modeling as well as simulation.

### 2.2 Modeling

In recent years, modeling has been under use as a powerful instrument, which at any time of filtration operations can detect the sites of incidence and mechanism of fouling. It can also help in adoption of the best decision for controlling fouling and optimizing the operational conditions. The models employed are categorized into two groups of individual models (a single mechanism) based on blocking laws (BL) and hybrid models (combined two-mechanism models) of fouling known as combined fouling (CM). The most practical models of the BL group include complete pore blocking (C), standard pore blocking (S), intermediate pore blocking (I), and cake formation (CF). The models of the CM subset also include intermediate pore blocking-standard pore blocking (I-S), cake formation-standard pore blocking (CF-S), cake formation-complete pore blocking (CF-C), cake formation-intermediate pore blocking (CF-I), as well as complete pore blocking-standard pore blocking (C-S) [30].

The fouling models have some key assumptions: 1) filtration occurs at constant pressure or flux, 2) membrane pores are cylindrical and uniform, and 3) depositing particles are uniform, non-deformable spheres. Each model has unique assumptions. Model C suggests fouling is a surface phenomenon where particles settle on surfaces without overlapping. Model I allows particles to settle on top of each other. Model CF describes deposits forming multiple layers, covering the entire membrane surface and increasing resistance proportionally with the cake layer's thickness. Model S assumes blockage happens inside the pores, reducing their diameter [29].

For the modeling to predict the quality of fouling (site and mechanism), the experimental data were matched against the models presented in Table 1 using Origin Pro 2022 v9.9.0.225 (SR1) software. The most common indices used for evaluating the models are mean squared error (MSE), root mean square error, sum squared error (SSE), coefficient of determination, mean absolute error (MAE), maximum absolute percentage error (Max APE), and mean absolute percentage error (MAPE). Among them, MSE and MAPE were set as the criteria of evaluation for choosing the suitable model (Eq. (1) and (2)).

(1) $$\text{Mean}\;\text{Squared}\;\text{Error}:\text{MSE}=\frac{1}{n}\;\sum_{i=1}^{n}{\left(y_{\text{iexp}}-y_{\text{ical}}\right)}^{2}$$

(2) $$\text{Mean}\;\text{Absolute}\;\text{Percentage}\;\text{Error}:\text{MAPE}\%=\frac{1}{n}\sum_{i=1}^{n}\frac{\left|y_{\text{iexp}}-y_{\text{ical}}\right|}{y_{\text{iexp}}}\times 100$$

Where n represents the total number of data, y_ical_ indicates the output data obtained from the model, and y_iexp_ denotes the experimental data. Note that by calculating the ratio of the coefficients of the models presented in Table 1 (K_c_J_o,_ K_b_/J_o_, K_i_, and K_s_ with 1/m unit), the share, as well as the predominant type of fouling mechanism, will be determined [51].

**Table 1:**
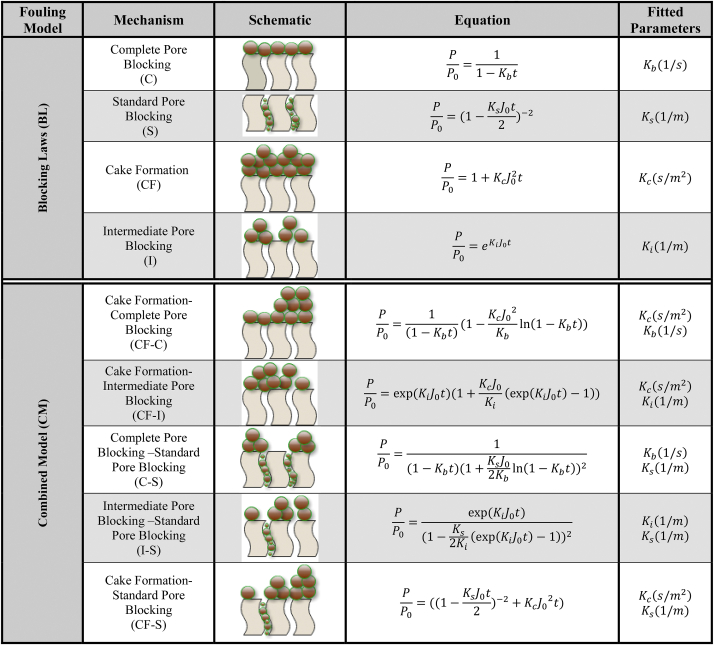
Equations of the Fouling model for constant flux operation.

### 2.3 Simulation

To predict the quantity of fouling (values of the cake and pore resistances along with the properties of the cake layer including its porosity, thickness, and mass), simulation was done via GPS-X 8.0 software using experimental data including temperature, aeration rate, membrane flux, HRT, and SRT. In this regard, TMP and mixed liquor suspended solid (MLSS) values acquired through simulation by software were compared to the actual experimental data to confirm the correct performance of the simulated system compared to the actual system. The parameters of mass, thickness, and resistance of the cake layer as well as the pore resistance were calculated by software according to Eqs. (3), (4), (5), (6).

(3) $$\frac{dm_{p}}{dt}=q_{perm}x_{Liq}f_{capture}-q_{backwash}x_{cake}f_{bw}-\left(\frac{q_{cross}}{A_{m}}\right)\left(\frac{x_{cake}}{x_{cake}+K_{S,cake}}\right)f_{cross}$$

(4) $$\delta_{C}=\frac{m_{p}}{\rho_{p}\left(1-\varepsilon \right)A_{m}}$$

(5) $$R_{C}=180\frac{\left(1-\varepsilon \right)m_{p}}{d_{p}^{2}\varepsilon^{3}A_{m}\rho_{p}}$$

(6) $$R_{f}=R_{fmax}\left(1-e^{-k_{f}t}\right)$$

In Eq. (3), the left term indicates the changes in the cake mass over time, while the right terms show bulk convection of solids to the surface of the membrane, the solids removed due to backwashing, and the solids removed due to crossflow aeration, respectively. In this equation, q_perm_, x_liq_, and f_capture_ indicate the permeate flow rate (m^3^/day), the concentration of solids in the bulk liquid phase within the MBR (kg/m^3^), and the solids' capture rate or fraction, respectively. Also, q_backwash_, x_cake_, and f_bw_ denote the backwash flow rate (m^3^/day), the mass of solids in the cake layer (kg/m^3^), and the backwash solids' removal rate (l/m^3^). Ultimately, q_cross_, A_m_, f_cross_, and K_S,cake_ indicate the crossflow or air scour flow rate (m^3^/day), total membrane surface area (m^2^), cross-flow solids' removal rate (kg/m), and half-saturation coefficient for cross-flow air (kg), respectively. In addition, in Eq. (4), $\delta_{c}$ denotes the cake layer thickness (m), $\rho_{p}$ shows the density of a cake layer particle (kg/m^3^), and ε represents the cake layer porosity. In Eq. (5), R_C_ shows the cake layer resistance (l/m) and d_p_ indicates the effective cake particle diameter (m). Ultimately, in Eq. (6), R_f_ shows the fouling resistance resulting from membrane pores (l/m), R_f,max_ indicates the maximum fouling resistance (l/m), k_f_ reflects the fouling rate constant (l/day), and t shows the time since last recovery clean. The default values of the software for the solids capture rate, the density of dry cake solids, maximum fouling resistance, and fouling rate constant were considered as 0.9999, 1.020 kg/m^3^, 1 × 10^12^, and 0.005 l/day respectively [52].

For all simulations, 1) the completely mixed MBR module from the Comprehensive – Carbon, Nitrogen, Phosphorus, pH (MANTIS2LIB) library was used; 2) The parameters of density and particle size of the cake layer were kept constant and equal to the optimal values of 1.02 × 106 mg/L and 1 μm, respectively for all simulations; 3) The MLSS was adjusted through the creation of SRT controller; 4) To investigate the impact of the density, porosity, and particle size of the cake layer, as well as the cross-flow rate on fouling, a sensitivity analysis was performed through simulation using the analyze menu.

## 3 Results and discussion

### 3.1 Investigating the extent of effectiveness of the membrane fouling reduction solutions in MBR

To determine the most effective solutions for reducing fouling, the factors affecting the membrane fouling in MBR were reviewed, with its results summarized in Table 2, Table 3, Table 4. The tables include the information related to the properties of wastewater and biomass (MLSS or mixed liquor volatile suspended solids (MLVSS), COD/N/P or C/N/P, organic loading rate (OLR), extracellular polymeric substances (EPS), feed to microorganism ratio (F/M)), membrane (material, size of pores, surface hydrophilicity), and operational conditions (HRT, SRT, temperature, aeration rate, flux, time). The results of each research contain percentage of COD removal as well as TMP of initiation and termination of MBR operation; and ultimately the cause and extent of fouling reduction. Note that the percentage of fouling reduction has been calculated by subtracting the difference between the initial and final TMP between the worst and best states of the operations divided by the difference of initial and terminal TMP in the worst state.

#### 3.1.1 Reduction of membrane fouling through modifying the properties of the inlet wastewater and biomass

The properties of wastewater and biomass play substantial roles in membrane fouling inside MBR including the C/N/P or COD/N/P, presence of salt, coagulants, and phenolic compounds in wastewater, pH, OLR, MLSS or MLVSS, EPS, soluble microbial products (SMPs), and F/M [53]. MLSS refers to the total amount of organic and inorganic suspended solids together with microorganisms in a mixed liquid, while MLVSS is part of organic materials belonging to volatile substances. Elevation of MLSS/MLVSS leads to easier deposition of sludge on the membrane surface, thus controlling it during the process seems essential. Further, OLR refers to the amount of organic materials consumed by the microorganisms inside MBR; with its elevation, due to an increase in the concentration of soluble organic materials on the surface of the membrane, the concentration polarization grows, and gradually a cake layer forms, and as such controlling OLR within a suitable range also appears essential [54]. In addition, changes in the C/N/P ratio through influence on factors such as biomass yield, MLSS, EPS, activated sludge hydrophobicity, and size of flocs, can affect the membrane fouling [55]. Meanwhile, the existence of chemicals such as NaCl salt through influencing COD, EPS, and SMP as well as changes in the microbial population [17], phenolic compounds [56], and coagulants such as alum via changing the size of flocs [15] can affect the membrane fouling. Finally, pH as an effective factor influencing COD and SMP can affect membrane fouling [14]. Table 2 reports the effect of each of the mentioned factors on reducing membrane fouling.

**Table 2:** The effect of properties of the inlet wastewater and biomass on reducing membrane fouling in MBR.

Row	Main Characteristics of Wastewater & Biomass			Main Characteristics of Membrane			Main Operational Condition				Result		Main Cause of Fouling Mitigation	Fouling Reduction (%)	Reference
	**MLSS(g/l)**	**COD/N/P**	**OLR** **(gCOD/L.day)**	**Polymer Type**	**pore size (μm)**	**Surface area (m**^**2**^**)**	**SRT (Day)**	**HRT** **(hour)**	**Flux** **(LMH)**	**Time (Day)**	**COD Removal (%)**	**TMP (Kpa)**			
												**In**			
	**MLVSS**	**C/N/P**										**Out**			
1	6.363	100/1.8/1	2.5	PVDF∗	0.3	0.03	15	24	10	130	98.40	4	COD/N/P increasing from 100/1.8/1 to 100/5/1	26	[55]
	NR∗	NR										9			
2	9.8	400/41/6	NR	PP∗	0.4	0.3	20	3	14.3	43	94.3	2.5	COD/N increasing from 5 to 10	83	[57]
	8.82	NR										35			
3	NR	NR	NR	PVDF	0.22	0.06	NR	12	5.21	75	95.5	0.9	C/N/P increasing from 100/5/1 to 100/10/1	80	[16]
		100/5/1										30.8			
4	14.3	NR	1.5–1.7	NR	0.4	0.2	20	31	NR	34	95	1.87	C/N increasing from 6/1 to 21/1	90	[58]
	6.15	21/1										8.86			
5	NR	NR	NR	PVDF	0.1	0.08	120	6	15	28	95	4	NaCl concentration decreasing from 40 to 0 g/l	85	[17]
												8			
6	5.1	150/5/1	0.25	PAN∗	0.1	0.2	40	8	2.5	110	NR	0.9	NaCl concentration decreasing from 35 to 0 g/l	50	[59]
	3.8	NR										0.16			
7	NR	NR	1.37	PEI∗	0.15	0.0278	NR	10	7.5	30	67	0.1	Phenolic compound concentration decreasing from 86 to 14 g/l	37	[56]
	2.89											0.4			
8	30.83	NR	NR	PE∗	0.4	0.2	30	NR	18.71	1 h	83.52	0.35	Coagulant addition (Alum)	52	[15]
	NR											1.15			
9	NR	NR	NR	CPE∗	0.2	0.116	200	6	15	1	98	2.95	pH control in neutral condition	50	[14]
	6											18			

NR: Not Reported.

PVDF: Poly-Vinyli Dene Fluoride.

PAN: Poly-Acrylo-Nitril

PEI: Poly-Ether-Imide.

PE: Poly- Ethylene.

CPE: Cholorinated Poly- Ethylene.

PP: Polypropylene.

According to Table 2, based on the study by Viero, de Melo [56] it was found that a reduction of the concentration of phenolic compounds has been able to stop TMP growth by up to 37 %. Meanwhile, Kunacheva, Soh [14] found that by controlling pH within the neutral range, the extent of TMP growth can be controlled by up to 50 %. Also, Wong, Ng [15] by adding alum observed a 52 % reduction in TMP growth. Elsewhere, the study by Chen, Hu [17] showed that a reduction of NaCl concentration from 40 to 0 mg/l led to an 85 % decrement in TMP growth. Ultimately, it can be stated that the overall elevation of the C/N/P ratio is associated with diminished membrane fouling; for example in the study by Erkan, Onkal Engin [58] with an increase in C/N ratio from 6 to 21, the extent of TMP growth along the operations dropped by up to 90 %. Therefore, the most important factors in controlling fouling seem to be ionic strength and the C/N/P ratio. These factors by adjusting MLSS, EPS, and SMP prevent the formation of a cake layer on the membrane surface and inhibit the increase of mass transfer resistance plus the pressure required for water to cross the membrane surface.

#### 3.1.2 Reduction of membrane fouling through modifying the membrane properties

Membrane constituent materials [60], hydrophilicity [12], superficial charge [61], roughness [62], pore size [63], specific surface area [64], and module structure are some examples of membrane characteristics that can prevent fouling. Common polymeric membranes include PE, polysulfone (PSf), polyethersulfone (PES), PAN, and PVDF, among which PVDF displays greater anti-fouling properties [53]. Furthermore, an increase in the surface hydrophilicity by forming a compressed hydration layer inhibits the development of fouling [65]. Meanwhile, the size of pores is one of the factors affecting fouling. In this regard, the smaller the size of membrane pores, the slower the increase in membrane pressure difference, and the greater the extent of cake formation on the membrane surface will be. On the other hand, larger membrane pore size is associated with increased membrane fouling because of greater blocking of pores. Thus, the size of pores should be optimized such that it minimizes both mechanisms of cake formation and pore blocking [53]. Table 3 indicates the effect of each of the mentioned factors on the reduction of membrane fouling.

**Table 3:** The effect of membrane properties on lowering the membrane fouling in MBR.

Row	Main Characteristics of Wastewater & Biomass		Main Characteristics of Membrane			Main Operational Condition				Result		Main Cause of Fouling Mitigation	Fouling Reduction (%)	Reference
	**MLSS(g/l)**	**EPS(mg/l)**	**Polymer Type**	**pore size (μm)**	**Surface area (m**^**2**^**)**	**SRT (Day)**	**HRT** **(hour)**	**Flux** **(LMH)**	**Time (Day)**	**COD Removal (%)**	**TMP (Kpa)**			
											**In**			
											**Out**			
1	4	164	CA∗	0.06	NR	15	NR	9.58	1 h	NR	2	Air contact angle increasing from 94 to 121° with the changing of membrane sub-layer from PVDF to CA	75	[18]
											5			
2	4.5	5.97	PVDF	0.0162	0.089	1.75	12	11	80	88	0	Water contact angle decreasing from 78 to 60° with go loading within membrane structure	67	[66]
											370			
3	4.5	1.74mgTOC/gSS∗	PVDF	0.1	0.012	30	16	25	30	93.5	0	Water contact angle decreasing from 87 to 79° with PTFE∗ blending with membrane structure	50	[67]
											30			
4	15	NR	PVDF	0.4	0.015	90	35	9.6	8.3	NR	3	Pore size increasing from 0.02 to 0.4 μm	84	[19]
											29			
5	0.51	NR	PES	0.2 KDa∗	0.031	30	22	10	80	97	8	Pore size increasing from 0.2 to 50 KDa	90	[68]
											35			
6	4.5	126.9 mg/g VSS∗	GO∗-CNC∗/PVDF	1.2	72	NR	NR	15	73	92	1	Hydrophilicity increasing with GO/CNC loading on the membrane surface	69.2	[69]
											40			

CA: Cellulose Acetate.

TOC: Total Organic Carbon.

SS: Suspended solids.

PTFE: Poly-Tetra-Fluoro-Ethylene.

KDa: Kilo-Dalton.

VSS: Volatile suspended solids.

GO: Graphen Oxide.

CNC: Cellulose Nano Crystal.

Based on Table 3, it is evident that an increase in surface hydrophilicity leads to membrane fouling reduction. In this regard, Jeon, Rajabzadeh [18] found that changing the membrane material from polymeric PVDF to ceramic CA is associated with enhanced hydrophilicity. This, in turn, could be followed by an extensive reduction of TMP growth during filtration (75 %). Elsewhere, by investigating two types of nanofiltration (NF) and ultrafiltration (UF) membranes, Tay, Liu [68] found that in NF, with small pore size, cake formation, and in UF with relatively large pores, pore fouling are predominant, confirming the effect of pore size on the mechanism of membrane fouling development. Therefore, adjustment of the membrane properties (such as porosity, number and size of pores, superficial functional groups, superficial charge, hydrophilicity, and hydrophobicity) is an effective solution for reducing fouling [70]. The most important membrane properties according to Table 3 are hydrophilicity and morphology of pores. Shrinkage of the size of pores by reducing the probability of entrapment of the contaminant inside the membrane pores can mitigate the pore fouling. However, it is followed by the accumulation of contaminants on the membrane surface and the formation of a cake layer. Meanwhile, an increase in the membrane hydrophilicity through adding hydrophilic modifiers to the membrane structure lowers the tendency of the nonpolar materials in the MBR medium to attach to the membrane surface, leading to reduced fouling [71]. Thus, if the membrane fabrication process can adjust the morphology of pores and enhance the surface hydrophilicity of the membrane concurrently, a minimal extent of fouling can be expected [72].

#### 3.1.3 Reduction of membrane fouling by modifying the operational properties

The operational parameters affecting the membrane fouling include SRT, HRT, temperature, flux, rate, and type of aeration, along with the duration of operations. SRT refers to the average time of residence of active solids in the bioreactor. It is suggested that for controlling fouling, sludge discharge be performed regularly. Also, HRT is the residence time of wastewater inside MBR, whose reduction can accelerate membrane fouling because of the elevation of the concentration of soluble organic materials [54]. The bioreactor temperature is another important operational factor whose control at optimal values leads to control of membrane fouling by influencing the values of SMP, EPS, polysaccharide (PS), proteins (PN), supernatant concentration, and turbidity [73]. It is also suggested that operational flux be controlled at values below the critical flux to prevent severe fouling of the membrane in MBR [65]. Aeration is one of the requirements of MBR which leads to the development of shear stress on the membrane surface and hence detachment of the cake layer, ultimately lowering the fouling [74]. Furthermore, regarding the size of air bubbles, it can be stated that coarse bubbles by reducing cake formation can control fouling [75]. Table 4 shows the effect of each of the above-mentioned factors on reducing membrane fouling.

**Table 4:** The effect of operational conditions on lowering the membrane fouling in MBR.

Row	Main Characteristics of Wastewater & Biomass		Main Characteristics of Membrane			Main Operational Condition						Result		Main Cause of Fouling Mitigation	Fouling Reduction (%)	Reference
	MLSS (g/l)	F/M (gCOD/gMLSS.d)	Polymer Type	pore size (μm)	Surface area (m2)	SRT (Day)	HRT (Hour)	Temperature (C)	Aeration Rate (L/min)	Flux (LMH)	Time (Day)	COD Removal (%)	TMP (Kpa)			
													In			
	MLVSS												Out			
1	NR	NR	ceramic	0.1	0.08	10	4	25–29	4	NR	24	93.3	1	Aeration with coarse bubbles instead of tiny bubbles	50	[75]
	NR												30			
2	10	NR	PE	0.15	0.4	50	7.2	NR	0.0417	4.5	27	NR	5	Using porous, flexible suspended carriers in MBR	90	[76]
	NR												30			
3	3	NR	PE	0.4	0.1	30	8	25	6	10	15	93.3	0	Using Attached Growth MBR (AGMBR)	42	[77]
	2.4												25			
4	5.4	NR	PVDF	0.2	0.1	23	6.8	20	6	8.82	12	NR	0	Using magnetic field coupled magnetic biochar MBR (MF-MB-MBR)	30	[78]
	NR												10			
5	10.1	NR	PV∗	0.4	0.5	30	6	25	12.5	20	10	NR	10	Using Submerged membrane bioreactor with granular sludge (Sub-MGSBR)	94	[4]
	NR												12			
6	0.09	0.91	PVDF	0.2	0.2	NS	18	NR	NR	8.33	75	97.4	2	HRT increasing from 6 to 18 h	85	[21]
	NR												37			
7	NR	0.5	CPE	0.2	0.116	200	4	35	8	15	6	95	3	HRT increasing from 1 to 4 h	27	[14]
	6												3.3			
8	5.2	NR	PE	0.4	0.11	25	24	27	7	1.89	50	99	0.9	HRT increasing from 18 to 24 h	60	[79]
	4.6												1.5			
9	21.5	NR	PES	0.2	0.98	40	2.2	26	NR	45	730	95	2.5	Temperature increasing from 8 to 26 °C	50	[73]
													10.5			
	15															
10	15	NR	PVDF	0.04	NR	20	17	27	NR	10	365	NR	5.2	Temperature increasing from 13 to 27 °C	55	[80]
	NR												22			
11	8–10	0.32	PES	0.2	0.065	NR	5.3	25	0.5	10	14	95	0	Temperature decreasing from 45 to 25 °C	91	[22]
													4			
	5.22															
12	10	NR	Zenon	NR	0.047	80	NR	20	NR	NR	190	94	0	SRT increasing from 20 to 80 day	40	[81]
	NR												29			
13	7.4	0.06	PVDF	0.22	0.3	NS∗	12	NR	4	9.7	90	90	1	SRT increasing from 10 to infinite day	85	[20]
	4.6												8			
14	10	0.13	NR	0.25	0.1	100	8	25	10	12.5	NR	98	0	SRT increasing from 20 to 100 day	70	[82]
	8.3												0.8			
15	21.9	NR	NR	0.4	NR	20	6	27	10	NR	155	94	3.8	SRT increasing from 3 to 20 day	66	[83]
	NR												14.48			
16	14	0.07	PE	0.4	0.3	70	12	25	20	42	0.083	97	15	SRT increasing from 30 to 70 day	26	[84]
	NR												28			
17	9.1	0.18	PVDF	0.1	0.02	30	8	25	1	11.12	50	95	2	SRT increasing from 10 to 30 day	85	[82]
	NR												35			
18	13.35	0.18	NR	0.4	20	20	24	NR	33–200	500	120	98	16	SRT increasing from 15 to 20 day	30	[85]
	10.29												20			

NS: No Sludge.

PV: Polyvinylidene.

According to Table 4, it is evident that the selection of suitable size of air bubbles is an effective factor in reducing fouling. In this regard, Zhao, Fu [75] found that the usage of coarse bubbles instead of fine bubbles would lower the TMP growth rate by up to 50 %. Also, Ibrahim, Sabeen [77] found that addition of cylindrical polythene media in MBR resulted in slow TMP increment (42 % reduction in TMP growth). Indeed, the collision between circulating media and hollow fiber led to frictional forces that mitigated cake formation on membrane. Furthermore, the use of porous and flexible suspended carriers in MBR led to membrane fouling mitigation. In this regard, the result of the study conducted by Yang, Chen [76] confirmed that suspended carriers in a state of three-section fluidity should affect the removal of cake layer in three ways; (1) the shear stress generated through suspended carriers scouring the membrane surface would remove the cake layer on the membrane surface; (2) the deposition of sludge particles would be moderated by means of the solute back-transport from the membrane surface due to the turbulence of suspended providers; (3) hollow-fiber membranes had been shaken through the effect of suspended carriers towards them, leading to removal of sludge particles deposited on the membrane surface. On the other hand, use of magnetic field coupled magnetic biochar MBR would have a positive effect on membrane fouling. In this regard, in the study conducted by Han, Jia [78], TMP growth dropped by 29 % because of enhancement of charge neutralization and the reduction of EPS formation, which yielded sludge flocs with a large pore structure conducive to form a fluffy and porous deposited layer in the membrane surface. Further, Wang, Li [4] found that use of submerged membrane bioreactor with granular sludge resulted in 94 % reduction in TMP growth because of strong shearing force induced by aeration with large granules with small flocs. In addition, control of temperature within the room temperature range would result in diminished fouling. Wang, Wu [73] by elevating the temperature from 8 to 26 °C observed a 50 % reduction in TMP growth, and Al-amri, Salim [22] by lowering the temperature from 45 to 25 °C, reported a 91 % reduction in TMP growth. Also, it can be stated that SRT elevation through an increase in MLSS and a decrease in F/M would lower the TMP growth. In this regard, as SRT increased from 10 to 30 days in the research by Yu, Yang [82], TMP growth dropped by 85 %. Meanwhile, enhancement of HRT through lowering the wastewater flow rate and decreasing F/M, results in diminished growth of microorganisms and ultimately decline of TMP growth. For example, in the study by Jiang, Ngo [21] as HRT increased from 6 to 18 h, TMP growth fell by 85 %. Accordingly, SRT and HRT by adjusting MLSS and F/M are the most important operational factors that reduce membrane fouling.

Based on the studies reviewed in this section, it was found that the most effective solutions for controlling and lowering membrane fouling inside MBR regarding adjustment of wastewater properties are controlling ionic strength and C/N/P ratio, while in the adjustment of membrane properties, they are hydrophilicity and pore size, and concerning adjusting operational conditions, they are HRT and SRT. Nevertheless, since adjustment of these factors requires the expenditure of costs, time, as well as technical knowledge, modeling, and simulation tools can be employed for the initial prediction of the membrane behavior in MBR in the long run under different conditions. Then, based on the obtained results, precise information about membrane fouling can be detected, and then with greater confidence, the necessary changes in the operating systems can be implemented. Therefore, for a more precise study of the mechanism and site of incidence of fouling as well as the properties of fouling formed during the process such as fouling resistances, thickness, mass, and porosity of the cake layer, modeling and simulation were performed on some studies, with the results presented in the subsequent sections.

### 3.2 Predicting the quality of fouling in MBR: site and mechanism

To know the site and mechanism of membrane fouling in MBR, modeling was performed on the data listed in some studies with the information mentioned in Section 2.2. The MSE and MAPE indices were evaluated as the criteria for choosing the best model (See Table 5). The details related to the other statistical indices completely are presented in Tables s-1-15.

**Table 5:** Selecting the best model describing the mechanism of fouling by MSE and MAPE parameters ∗ The best chosen model has been specified by bold and Italic font.

Research Conducted By		C		S		CF		I		CF-C		CF-I		C-S		I-S		CF-S	
		MSE (∗10^−5^)	MAPE	MSE (∗10^−5^)	MAPE	MSE (∗10^−5^)	MAPE	MSE (∗10^−5^)	MAPE	MSE (∗10^−5^)	MAPE	MSE (∗10^−5^)	MAPE	MSE (∗10^−5^)	MAPE	MSE (∗10^−5^)	MAPE	MSE (∗10^−5^)	MAPE
Wang, Li [4]		3.01	4.68	1.56	4.53	33.96	21.47	9.58	11.27	FM	FM	7.09	9.71	FM	FM	1.56	4.53	***0.48∗***	***1.72***
Erkan, Onkal Engin [58]		0.691	6.15	0.498	4.63	5.76	15	1.42	7.12	0.868	5.69	1.08	6.27	0.498	4.63	0.51	4.64	***0.49***	***4.72***
Ibrahim, Sabeen [77]		404.29	105.44	11.08	30.81	35.29	33.27	6.31	19.15	3.48	17.5	4.97	19.44	FM	FM	4.25	19.64	***1.66***	***11.2***
Ouyang and Liu [20]		3.61	14.4	1.96	17.4	9.08	38.5	3.31	24.6	2.04	17.3	2.78	22.7	1.95	16.9	1.96	17.4	***1.83***	***14.5***
Feng, Zhang [57]		14.85	8.91	19.98	13.8	228.3	40.1	85.06	25.34	FM	FM	66.68	22.8	FM	FM	19.98	13.77	***5***	***7.65***
Wang, Wu [73]		17.4	7.59	15.8	9.8	81.7	28	33.1	17.5	13.5	6.81	27.8	15.7	13.5	6.74	15.8	9.8	***13.2***	***6.06***
Fallah, Bonakdarpour [79]		1.78	21.4	1.52	19.4	0.956	15.6	1.29	17.9	0.627	14.8	0.609	13.4	1.52	19.4	***0.571***	***13.3***	0.956	15.6
Han, Jia [78]		470.82	100	96.17	45.22	8.08	33.91	26.82	24.36	2.79	19.79	2.56	18.66	FM	FM	***1.26***	***7.76***	4.3	25.17
Yu, Yang [82]	**Part1**	***1.42***	***10.9***	1.97	12	8.46	23.6	3.85	14.8	1.34	11.2	3.32	13.8	1.35	11.2	1.97	12	1.61	11.8
	**Part2**	188	24.2	196	23.8	211	23	202	23.5	183	24.5	200	23.5	***12.5***	***8.93***	196	23.8	98.4	16.7
Zhao, Fu [75]		2600	101	267	43.7	210	44.9	4.6	12.1	***4.26***	***8.78***	4.58	12.1	267	43.7	4.58	12.1	19	12.8
Tay, Liu [68]		3670	110	61.5	11.4	142	16.3	87.4	13.2	***38.3***	***10***	80.7	12.7	38.2	10.2	61.5	11.4	50.8	11.5
Miyoshi, Yuasa [19]		2720	94.1	1890	86.2	***37.8***	***19.8***	763	65.9	2440	91.3	448	56	1890	86.2	309	49.2	37.9	19.8
Babatsouli, Palogos [85]		595.67	115.45	38.74	19.69	20.58	28.42	12.61	14.18	9.43	17.31	***8.91***	***16.47***	FM	FM	561.75	94.8	10.94	19.3
Yang, Chen [76]		25.4	26.78	8.75	15.85	4.35	14.54	1.11	5.45	0.41	4.7	***0.22***	***3.04***	FM	FM	0.22	3.22	0.62	5.81

FM: Failed Modeling.

According to Table 5, the best model was chosen based on MSE and MAPE evaluation. The research by Babatsouli, Palogos [85] and Yang, Chen [76], with the CF-I model matched MSE of 8.91 × 10^5^ and 0.22 × 10^5^ respectively. This indicates the accumulation of particles in the form of multilayers on the surface of the membrane pores without their complete blockage and finally the formation of a permeable cake layer. In the studies by Ouyang and Liu [20], Erkan, Onkal Engin [58], Wang, Wu [73], Feng, Zhang [57], Ibrahim, Sabeen [77] and Wang, Li [4], they had a greater match with the CF-S model with MSE values of 1.83 × 10^5^, 0.49 × 10^5^, 13.2 × 10^5^, 5 × 10^5^, 1.66 × 10^5^, and 0.48 × 10^5^ respectively. This demonstrates diffusion of the contaminant particles concurrently inside the membrane pores as well as aggregation on the membrane surface and formation of the cake layer. Also, in the studies by Tay, Liu [68], Zhao, Fu [75], MSE values obtained as 38.3 × 10^5^ and 4.26 × 10^5^ were matched against the CF-C model. It can be concluded that the contaminant particles through deposition on the membrane surface would lead to the blockage of membrane pores as a single layer as well as the formation of a cake layer on the membrane surface as a multilayer. In addition, the research by Fallah, Bonakdarpour [79] and Han, Jia [78], with the I-S model was matched with MSE of 0.571 × 10^5^ and 1.26 × 10^5^ respectively, suggesting aggregation of contaminant particles inside the pores and gradual shrinkage of membrane pore size. Also, the research by Miyoshi, Yuasa [19] with an MSE of 37.8 × 10^5^ matched the CF model, indeed predicting that most contaminant particles under the available operational conditions are larger than the membrane pores. Thus, because of the impossibility of entrance to the pores, contaminant particles accumulate as multilayers on the membrane surface, and eventually a cake layer is formed. In some occasions, fouling is a complex process, whose mechanism of development changes over time [86]. In this respect, modeling of experimental data presented by Yu, Yang [82] showed the development of the fouling mechanism based on complete pore blocking at initial moments of filtration (through correspondence with Model C with MSE 1.42 × 10^5^), followed by its change to standard and complete blockage of pores mechanisms over time (through matching against C-S model with MSE of 12.5 × 10^5^). Possibly, the initial aggregation of contaminant particles on the surface of pores as single-layer results in complete pore blockage. However, over time, the particles deposited on the surface of pores, because of an increase in the operational pressure along the process, enter the membrane pores causing narrowing of the pore walls. Ultimately, according to Table 5, the best model describing the mechanism and site of incidence of fouling was specified. Then, K_c_(s/m^2^), K_s_(1/m), K_i_(1/m), and K_b_(1/s) coefficients were obtained through modeling for the best model. Thereafter, to determine the predominant mechanism of fouling in the combined fouling models, by knowing the flux, K_i_/K_s_, K_c_J_0_/K_s_, and K_c_J_0_^2^/K_b_ ratios were calculated, with the results presented in Table 6.

**Table 6:** Determining the predominant mechanism of fouling through investigating the modeling coefficients.

Research Conducted By	Model	K_c_(s/m^2^) (∗10^−5^)	K_S_(1/m) (∗10^+1^)	K_i_(1/m) (∗10^+1^)	K_b_(1/s) (∗10^+7^)	J_0_ (m^3^/m^2^s) (∗10^+7^)	K_i_/K_s_	K_c_J_0_/K_s_	K_c_J_0_/K_i_	K_c_J_0_^2^/K_b_
Wang, Li [4]	CF-S	−0.11	1.7	–	–	55.6	–	0.35	–	–
Erkan, Onkal Engin [58]	CF-S	−157	888	–	–	8.961	–	0.158	–	–
Ibrahim, Sabeen [77]	CF-S	5.05	4.4	–	–	27.8	–	3.19	–	–
Ouyang and Liu [20]	CF-S	−0.0528	0.566	–	–	26.9	–	0.251	–	–
Feng, Zhang [57]	CF-S	−0.1777	0.92	–	–	39.72	–	0.76	–	–
Wang, Wu [73]	CF-S	−0.0159	0.221	–	–	125	–	0.899	–	–
Fallah, Bonakdarpour [79]	I-S	–	−2.96	11.6	–	5.25	3.92	–	–	–
Han, Jia [78]	I-S	–	−0.04	1.62	–	24.5	35.8	–	–	–
Zhao, Fu [75]	CF-C	10.5	–	–	3.64	26.9	–	–	–	20.87
Tay, Liu [68]	CF-C	−0.264	–	–	4.11	27.8	–	–	–	0.496
Babatsouli, Palogos [85]	CF-I	0.046	–	0.062	–	57.9	–	–	4.32	–
Yang, Chen [76]	CF-I	−0.8	–	26.1	–	12.5	–	–	0.04	–

According to Table 6, in the research by Fallah, Bonakdarpour [79]and Han, Jia [78], the value of K_i_/K_S_ has been equal to 3.92 and 35.8 respectively, indicating a larger share of the intermediate pore blocking compared to standard pore blockage. In addition, in the research by Babatsouli, Palogos [85] according to Table 6, the value of K_c_J_0_/K_i_ has been equal to 4.32, indicating a larger share of cake formation compared to intermediate pore blockage while in the research by Yang, Chen [76], the value of K_c_J_0_/K_i_ has been equal to 0.04 and indicating a larger share intermediate pore blockage compared to cake formation. Also, the K_c_J_0_/K_s_ value according to the research by Ouyang and Liu [20], Erkan, Onkal Engin [58], Wang, Wu [73], Feng, Zhang [57], and Wang, Li [4], was found 0.251, 0.158, 0.899, 0.76, and 0.35 respectively, suggesting the larger share of the standard pore blockage model compared to cake formation. On the other hand, the K_c_J_0_/K_s_ value according to the research by Ibrahim, Sabeen [77], was found 3.19, suggesting the larger share of the cake formation model compared to standard pore blockage. In the research by Tay, Liu [68] according to Table 6, the K_c_J_0_^2^/K value has been smaller than one and equal to 0.496, indicating a larger contribution to complete pore blockage compared to cake formation. Meanwhile, since in the research by Zhao, Fu [75], the K_c_J_0_^2^/K ratio has been larger than one and equal to 20.87, the cake formation claims a larger share compared to complete pore blockage in membrane fouling creation. Investigation of the experimentally measured resistances in this research and the larger cake resistance compared to other resistances confirms the above result. Overall, it can be stated that modeling has not only been able to present the best mechanism describing fouling during the MBR process (according to Table 5) but also according to Table 6, in the combined fouling models, it is also able to determine the predominant mechanism of fouling. Meanwhile, knowing the predominant mechanism of fouling can provide the MBR user with suitable solutions for controlling fouling. For example, in the research by Tay, Liu [68], where complete pore blocking has a more prominent role compared to cake layer formation in fouling, it is better to use chemical washing at a suitable time to eliminate the fouling of pores [53]. However, in the research by Zhao, Fu [75] where the role of cake layer formation has been more prominent in the development of fouling, solutions such as the use of baffle, spacer, and high-speed aeration for creating turbulence and forming shear flow on the membrane surface can be used to prevent cake layer formation [87]. Next, to determine the quantity of fouling (precise values of the resistances of cake and pore along with thickness, porosity, and cake layer mass), simulation has been performed via GPS-X software, with its results presented further.

### 3.3 Predicting the quantity of fouling in MBR: properties and resistances of cake layer and pore

For determining the quantity of fouling including the precise values of cake and pore resistances along with the thickness, porosity, and cake layer mass in the target studies, simulation was performed by GPS-X software according to the method described in Section 2, 3. The input parameters for simulation alongside their values as well as the correspondence of experimental data with the simulated system are presented in Table 7.

**Table 7:** The input parameters of simulation alongside correspondence of the results obtained from simulation against experimental data.

Research Conducted By	Input Parameters For Simulation					Fitting Experimental Data with Simulation					
	SRT (days)	HRT (h)	Flux (L/m^2.^h)	Aeration Rate (L/min)	Temperature (°C)	TMP (kPa)			MLSS (g/L)		
						Experiment	GPS-X	MSE	Experiment	GPS-X	MSE
Wang, Li [4]	30	6	20	12.5	25	10.08–51.25	10.63–50.99	1.54	7	6.78–7.46	0.38
Erkan, Onkal Engin [58]	20	31	3.226	1.8	20	1.88–8.86	1.59–8.86	0.33	14.3	14.19–14.34	0.005
Ibrahim, Sabeen [77]	30	8	10	6	25	2.67–41.44	1.28–41.6	4.78	1.9–3.7	3	0.3
Ouyang and Liu [20]	NS	12	9.7	4	20	1.34–6.82	1.20–7.1	0.08	7.4	2.61–7.4	2
Feng, Zhang [57]	20	3	14.3	3.33	25	3.67–30.79	4.37–30.62	0.23	9.8	8.92–10.67	2.99
Wang, Wu [73]	10–40	2.2	45	4800	11–26	3.44–12.33	3.78–17.21	2.57	4.72–21.51	3.12–18.97	6.37
Fallah, Bonakdarpour [79]	9	24	1.89	7	25	0.32–1.16	0.32–1.54	0.23	4.26–6.36	4.71–5.67	0.13
Han, Jia [78]	23	6.8	8.82	6	20	0.78–35.88	1.7–35.22	0.95	5.1–5.5	5.4	0.32
Yu, Yang [82]	30	8	11.12	1	25	1.54–34.42	0.85–35.85	1.4	8.623	1.72–8.9	9.59
Zhao, Fu [75]	10	4	9.7	4	27	1.23–29.81	0.95–30.76	0.43	2.26–4.48	4.5	1.39
Tay, Liu [68]	30	22	10	3	20	7.72–35.85	6.92–33.31	6.91	0.503	0.0–0.54	0.04
Miyoshi, Yuasa [19]	90	35	9.6	2	20	2.86–28.36	1.09–29.59	3.39	15	14.74–15.81	0.11
Babatsouli, Palogos [85]	20	24	500	33–200	25	4.66–39.83	5.18–39.14	1.85	25	21.75–25.67	5.65
Yang, Chen [76]	50	7.2	4.5	0.0417	25	4.57–29.67	6.93–30.33	1.47	10	9.12–12.14	2.25

**Fig. 1:**
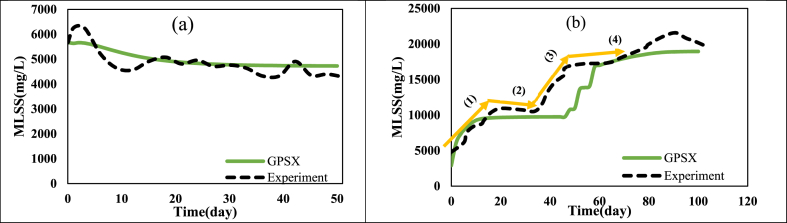
Correspondence between experimental data and those obtained from simulation related to MLSS parameter in studies by: a) Fallah, Bonakdarpour [79] and b) Wang, Wu [73].

According to Table 7, it is evident that the results of the simulation of the target studies have an acceptable match with real data; the MSE value for the TMP data has been at most 6.91 (kPa^2^), while for the data related to MLSS, it has been at most 9.59 (g^2^/l^2^). Meanwhile, the success of simulation is attributed to a wide range of parameters. SRT value has been considered at around 9 up to infinite days, HRT between 2.2 and 35 h, flux between 1.89 and 500 LMH, aeration rate between 0.0417 and 4800 l/min, and temperature from 11 to 27 °C. In addition, studies by Miyoshi, Yuasa [19], Ouyang and Liu [20], Erkan, Onkal Engin [58], Tay, Liu [68], Zhao, Fu [75], Fallah, Bonakdarpour [79], Yu, Yang [82], Ibrahim, Sabeen [77], Wang, Li [4], Han, Jia [78], Feng, Zhang [57], Yang, Chen [76], were all on a laboratory scale, while the research by Wang, Wu [73], Babatsouli, Palogos [85], were on a pilot scale. Accordingly, it can be stated that simulation by software can be done within a wide scale range (experimental, pilot, and industrial) as well as input parameters (SRT, HRT, Flux, Aeration rate, Temperature), thereby leading to reliable results. Fig. 1 presents the correspondence between experimental and simulation data related to MLSS parameter for the studies conducted by Wang, Wu [73], Fallah, Bonakdarpour [79]. Note that in other examined studies, MLSS value over time has been constant and according to the numbers reported in Table 7.

Based on Fig. 1, it is evident that the simulation has well been able to predict MLSS variations over time. Indeed, it has even been able to predict leaps and irregularities in MLSS trends along the process. According to Fig. 1-b, experimental data indicate gradual rise of MLSS on the initial days (Change No. 1), followed by reaching an initial steady-state (change No. 2), gradual regrowth of MLSS (change No. 3), and ultimately reaching a secondary steady-state (change No. 4). This is due to performing the operations at SRT of 10 days in the initial days and then elevation of SRT on the forty-fourth days of the process to 40 days. Indeed, during the initial days of the process with SRT = 10 days, due to sludge feeding, biomass grows, causing an initial rise of MLSS. Then, its value reaches a stable value of 10–11 g/l, and during the middle days of the process when the SRT rises to 40 days, MLSS increases again, eventually reaching a secondary steady value of 20–22 g/l. As observed in Fig. 1-b, through predicting SRT variations along the simulation, MLSS changes have also been well predicted. Ultimately, Table 8 reports the results related to the quantity of fouling including the precise values of cake and pore resistances along with thickness, porosity, and cake layer mass, which would help the researcher predict the behavior of the actual system more accurately.

**Table 8:** Determining the quantity of fouling through simulation by GPS-X software.

Research Conducted By	Pore Resistance ∗ 10^−11^ (1/m)	Cake Resistance ∗ 10^−11^ (1/m)	Cake Porosity (%)	Cake Thickness (μm)	Cake Mass (g)
Wang, Li [4]	40.6	43.9	32–80	1728	599
Erkan, Onkal Engin [58]	0.00868	88.7	11.8–40	104.1	18.73
Ibrahim, Sabeen [77]	2.42	166	26–39.5	2950	222.7
Ouyang and Liu [20]	3.62	16.5	54–71	6804	957.7
Feng, Zhang [57]	0.007	74.4	31–75	2685	564
Wang, Wu [73]	3.94	13	67–75	17910	130400
Fallah, Bonakdarpour [79]	2.21	21.9	8–27	7.36	0.76
Han, Jia [78]	12.6	132	12.8–40	202	18
Yu, Yang [82] Part 1 and Part 2	0.209	33.8	29–35	900	12.91
	2.29	104	21–29	850	13.79
Zhao, Fu [75]	18.8	166	13–37	267.5	18.99
Tay, Liu [68]	5.88	95.9	24.4–37.5	1355	32.38
Miyoshi, Yuasa [19]	0.392	111	9.7–19.1	68.78	1.901
Babatsouli, Palogos [85]	6.32	26.5	82–90	250500	1984000
Yang, Chen [76]	1.96	152	12–30	567	193

**Fig. 2:**
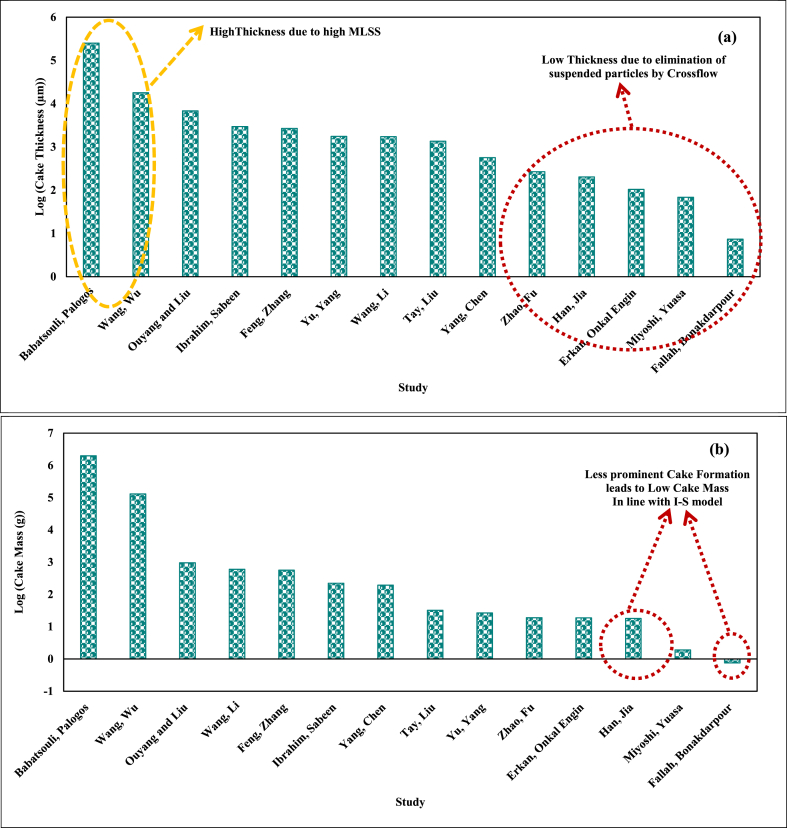
a) Log (Cake Thickness) and b) Log (Cake Mass) in simulated studies.

According to Table 8, it is observed that the thickness of the cake formed in the research by Miyoshi, Yuasa [19], Erkan, Onkal Engin [58], Zhao, Fu [75], Fallah, Bonakdarpour [79], Han, Jia [78], has been relatively lower (68.78, 104.1, 267.5, 7.36, and 202 μm, respectively) compared to other examined studies (see Fig. 2 a). This can be due to a lack of aggregation and considerable elimination of suspended particles on the membrane surface caused by the crossflow, causing the formation of a thinner cake layer. Also, based on Table 7, it is seen that the research by Wang, Wu [73] has reported the minimum HRT (2.2 h) and relatively higher MLSS (21.51 g/l) compared to other investigated studies. In this research, on the one hand, low HRT values have caused elevation of OLR, F/M, growth of microorganisms, and SMP, and on the other hand, high MLSS values have resulted in an increase of suspended solids and dead microorganisms along with reduced dissolved oxygen (DO) concentration. Overall, these factors are associated with increased fouling. By investigating the results of the quantity of fouling obtained from simulation according to Table 8, it is seen that the thickness (17910 μm) and mass (130400 g) of the cake layer formed in the research by Wang, Wu [73] have been higher compared to other examined ones except the research conducted by Babatsouli, Palogos [85]. Indeed, the maximum MLSS (25 g/l) in the research by Babatsouli, Palogos [85] led to the maximum cake thickness (250500 μm) and mass (1984000 g) (see Fig. 2 a). Thus, while confirming the described mechanism, the important role of simulation in the proper prediction of the results is proven. Meanwhile, the minimum thickness (7.36 μm) and mass (0.76 g) of the cake layer according to Table 8, are related to the research by Fallah, Bonakdarpour [79], thereby indicating the less prominent role of fouling resulting from cake layer compared to pore fouling. This is also in line with the results obtained from the modeling. Indeed, this study matched the I-S model (based on Table 5). This finding can also be generalized to the research conducted by Han, Jia [78], as in this research, the mass (18 g) and thickness (202 μm) of the cake layer have been relatively low, and according to Table 5, the result in Table matches model I-S (see Fig. 2 b).

**Fig. 3:**
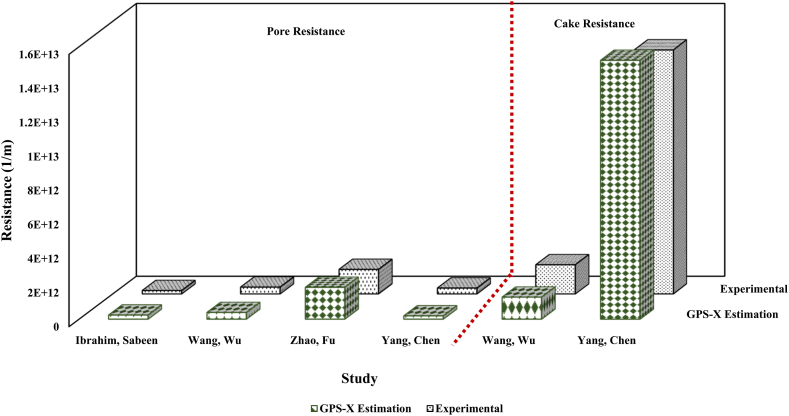
Values of experimental and predicted pore and cake resistances by the GPS-X and their comparison.

In addition, modeling of the research by Zhao, Fu [75] indicated a greater share of the cake formation mechanism compared to the complete pore blocking mechanism in developing fouling (correspondence with CF-C model) (according to Table 6). This is also confirmed by the results obtained from the simulation. Indeed, the cake resistance (166 × 10^+11^ 1/m) is around 10 times larger than the pore resistance (18.8 × 10^+11^ 1/m). Meanwhile, according to the modeling results (section 3-2), it was found that the research by Miyoshi, Yuasa [19] matches the CF individual model, suggesting the more prominent role of fouling resulting from cake layer formation compared to other mechanisms of fouling including pore resistance. This was also confirmed by simulation; based on Table 8, the cake resistance value (111 × 10^+11^ 1/m) is around 1000 times greater than the pore resistance (0.392 × 10^+11^ 1/m). Specifically, according to Table 8, the cake resistance (166 × 10^+11^ 1/m) value is around 68 times greater than the pore resistance (2.42 × 10^+11^ 1/m) for the research conducted by Ibrahim, Sabeen [77]. This can be attributed to the prominent role of fouling resulting from cake layer formation verified by modelling results as well. Indeed, in this research, K_c_J_0_/K_s_ is higher than one (3.19 according to Table 6), demonstrating the larger share of the cake formation model compared to standard pore blocking. Also, Fig. 3 shows the values of experimental and predicted pore and cake resistances by the GPS-X and their comparison. It should be mentioned that studies reporting the experimental values of resistances are included in Fig. 3.

Fig. 3 displays the appropriate accuracy of the GPS-X in predicting the cake and pore resistance values. For example, it can be mentioned that the pore resistance estimated by the GPS-X is equal to 2.42 × 10^+11^ 1/m, and this value is reported to be equal to 2 × 10^+11^ 1/m in the research conducted by Ibrahim, Sabeen [77]. In addition, in the research conducted by Wang, Wu [73], the resistances caused by microbial flocs and solutes have been 1.72 × 10^+12^ 1/m and 4.01 × 10^+11^ respectively. According to Table 8 and Fig. 3, R_c_ and R_p_ values are equal to 1.3 × 10^+12^ 1/m, and 3.94 × 10^+11^ 1/m respectively. Indeed, microbial flocs which accumulate on membrane surface lead to cake formation on the membrane surface, and solutes that cross the membrane pores cause pore resistance. Meanwhile, the resistance values reported in the research and obtained from the GPS-X are consistent with good accuracy, which confirms the accuracy of the simulation.

Overall, it can be stated that simulation by GPS-X software, in addition to operability within a wide range of input parameters as well as the ability to properly prediction of the trend of changes in MLSS and TMP, can well calculate important quantities of fouling including pore and cake resistances plus the properties of the formed cake layer such as mass, porosity, and thickness. The minimum pore resistance was related to research by Feng, Zhang [57], and Erkan, Onkal Engin [58] (0.007 × 10^+11^ 1/m and 0.00868 × 10^+11^ 1/m, respectively according to Table 8). In these studies, the solution for reducing fouling was increasing the COD/N ratio from 5 to 10, and C/N ratio from 6.1 to 21.1 respectively (see Table 2). Thus, it seems that adjustment of the COD/N and C/N/P ratio can have a remarkable impact on the pore resistance value and lower the fouling that results from it. It is also observed that the minimum cake resistance (13 × 10^+11^ 1/m and 26.5 × 10^+11^ 1/m) is related to the research by Wang, Wu [73] as well as Babatsouli, Palogos [85] (according to Table 8). Meanwhile, the thickness (17910 and 250500 μm) and mass (130400 and 1984000 g) of the cake layer formed in these researches have been far larger compared to other studies, due to the formation of a far porous cake layer ((67–75 %) and (82–90 %)). Meanwhile, the largest cake resistance (166 × 10^+11^ 1/m, 166 × 10^+11^ 1/m, and 111 × 10^+11^ 1/m) and relatively lowest cake layer porosity ((26–39.5 %), (13–37 %) and (9.7–19 %)) are related to studies by Ibrahim, Sabeen [77], Miyoshi, Yuasa [19], Zhao, Fu [75], respectively (Table 8). Thus, it seems that in MBR processes, the formation of a cake layer with high porosity, despite the high thickness and mass, not only fails to increase mass transfer resistance for water crossing but can also function as an auxiliary membrane and maintain a high level of separation.

### 3.4 The status of modeling and GPS-X simulation of MBR systems and the comparison with the current research

In order to present the status of the current research, other studies conducted in the field of modeling and GPS-X simulation of MBR systems were reviewed with the results provided in Table 9.

**Table 9:**
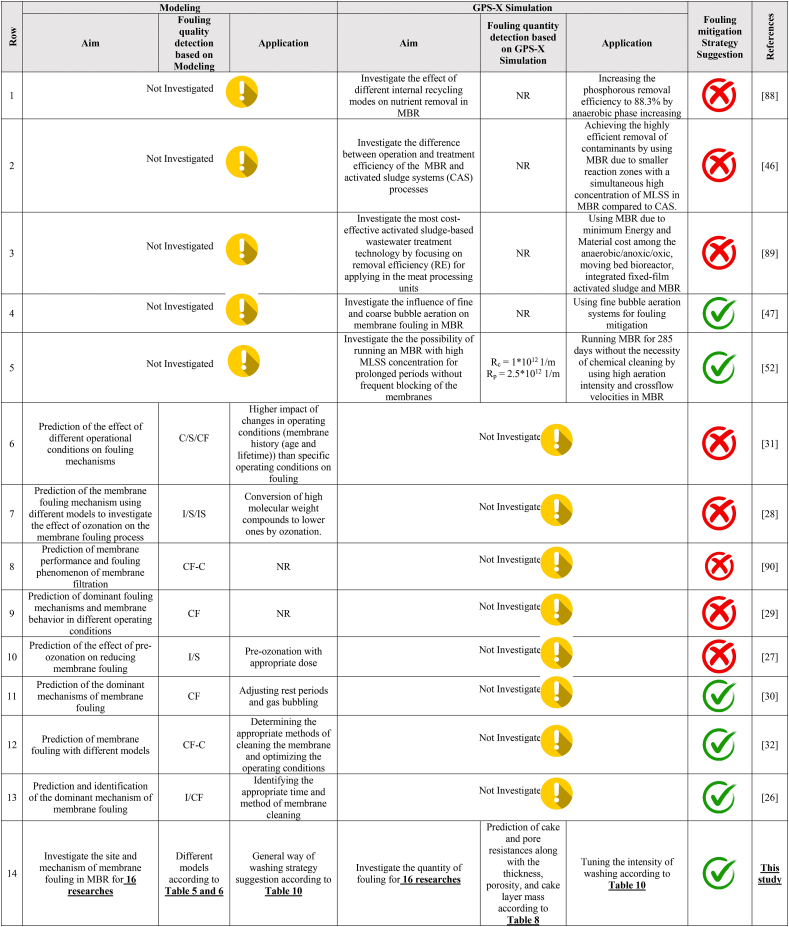
Comparison of the results of this research with other studies on modeling and GPS-X simulation of MBR systems [[88], [89], [90]].

**Table 10:** Presenting the best strategy of controlling fouling along the operations based on the modeling and simulation results.

Research Conducted By	Main Strategy in Research at Initial Time	Fouling Mechanism Model		Resistance ∗ 10^−11^ (1/m)				Suggested Strategy at Filtration Time					
				Pore		Cake		Physical			Chemical		
								High	Medium	Low	High	Medium	Low
Wang, Li [4]	Using Submerged membrane bioreactor with granular sludge (Sub-MGSBR)	CF-S		40.6		43.9			∗		∗		
Erkan, Onkal Engin [58]	C/N increasing from 6/1 to 21/1	CF-S		0.00868		88.7			∗				∗
Ibrahim, Sabeen [77]	Using Attached Growth MBR (AGMBR)	CF-S		2.42		166		∗				∗	
Ouyang and Liu [20]	SRT increasing from 10 to infinite day	CF-S		3.62		16.5				∗		∗	
Feng, Zhang [57]	COD/N increasing from 5 to 10	CF-S		0.007		74.4			∗				∗
Wang, Wu [73]	Temperature increasing from 8 to 26 °C	CF-S		3.94		13				∗		∗	
Fallah, Bonakdarpour [79]	HRT increasing from 18 to 24 h	I-S		2.21		21.9				∗		∗	
Han, Jia [78]	Using magnetic field coupled magnetic biochar MBR (MF-MB-MBR)	I-S		12.6		132		∗			∗		
Yu, Yang [82] Part 1/Part 2	SRT increasing from 10 to 30 day	C	C-S	0.209	2.29	33.8	104			∗		∗	
Zhao, Fu [75]	Aeration with coarse bubbles instead of tiny bubbles	CF-C		18.8		166		∗			∗		
Tay, Liu [68]	Pore size increasing from 0.2 to 50 KDa	CF-C		5.88		95.9			∗			∗	
Miyoshi, Yuasa [19]	Pore size increasing from 0.02 to 0.4 μm	CF		0.392		111		∗					
Babatsouli, Palogos [85]	SRT increasing from 15 to 20 day	CF-I		6.32		26.5				∗		∗	
Yang, Chen [76]	Using porous, flexible suspended carriers in MBR	CF-I		1.96		152		∗				∗	

Table 9 presents the research results on modeling (based on series resistance theory) and simulating (using GPS-X software) MBR systems. The primary objective of most studies in the field of GPS-X simulation has been to improve removal efficiency in MBR systems. Few simulations have focused on reducing membrane fouling, and these have not investigated the quality or characteristics of the fouling layer, such as mass and thickness. Conversely, numerous studies have modeled various objectives, including predicting dominant fouling mechanisms, forecasting membrane fouling using different models, and assessing the impact of operational conditions on fouling mechanisms. Some studies have proposed strategies for fouling reduction, such as adjusting relaxation periods, gas sparging, optimizing membrane cleaning methods, and determining the appropriate timing as well as method for membrane cleaning. However, none of these studies have quantitatively assessed fouling. This research examines all factors influencing fouling, determines the quality of fouling through modeling, and quantifies fouling using GPS-X simulation. It provides suggestions for reducing membrane fouling across 14 studies.

### 3.5 Presenting solutions for controlling fouling during the operations based on the modeling and simulation results

**Fig. 4:**
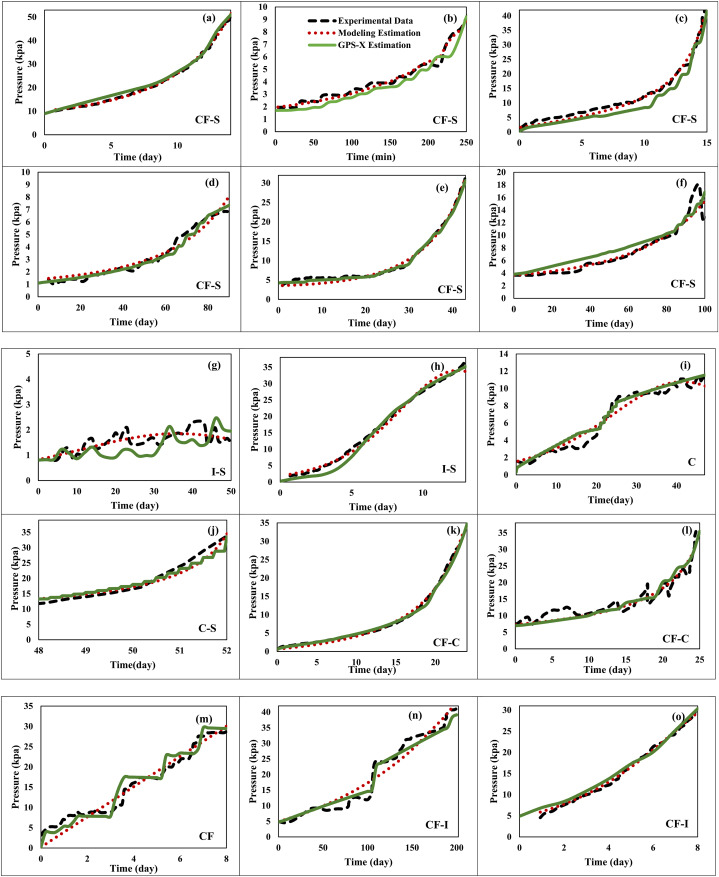
The match between experimental, modeling, and simulation results related to the TMP parameter in the studies by a) Wang, Li [4], b) Erkan, Onkal Engin [58], c) Ibrahim, Sabeen [77], d) Ouyang and Liu [20], e) Feng, Zhang [57], f) Wang, Wu [73], g) Fallah, Bonakdarpour [79], h) Han, Jia [78], i) and j) Yu, Yang [82]-part 1 and 2, k) Zhao, Fu [75], l) Tay, Liu [68], m) Miyoshi, Yuasa [19], n) Babatsouli, Palogos [85], and o) Yang, Chen [76].

As stated earlier, in MBR systems one of the most important problems is the development of membrane fouling. Thus, to save energy consumption and costs, in addition to the solutions considered at the beginning of the process, some solutions should also be applied along the operations periodically. To present the best solution for controlling fouling along the operations, first, it is better to identify the trend of TMP variations. For this purpose, Fig. 4 indicates a match between experimental, modeling, and simulation results.

According to Fig. 4, in the studies with long operational times such as the ones by Ouyang and Liu [20], Wang, Wu [73], (Fig. 4d–f and n), the prediction of the fouling models has proved to be more accurate in comparison to the studies with short operational times such as the ones by Miyoshi, Yuasa [19], Erkan, Onkal Engin [58], Tay, Liu [68], (Fig. 4, b, c, g, h, i, j, l, and m). This can be due to the reduction of TMP variations with prolongation of the timescale. In addition, fouling models are in the form of exponential equations that predict a specific ascending or descending trend for TMP variations (according to Table 1). On the other hand, TMP variations in an actual system can experience minor fluctuations under the influence of the distribution and density of the cake layer along with aeration. Accordingly, the results obtained from the simulation while considering the changes in porosity and thickness of the cake layer show a closer behavior to the actual system (Fig. 4a–e, k, and o).

**Fig. 5:**
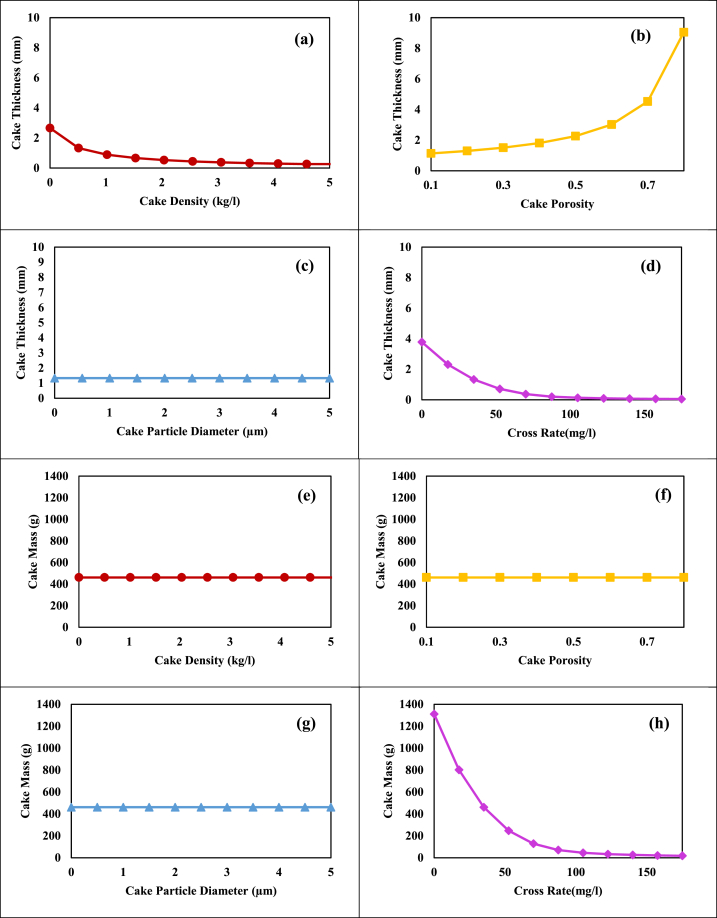
The sensitivity analysis results for the effects of: a, e) Cake density; b, f) Cake porosity; c, g) Cake particle diameter; d, h) Crossflow rate on the thickness and mass of the cake layer, respectively.

**Fig. 6:**
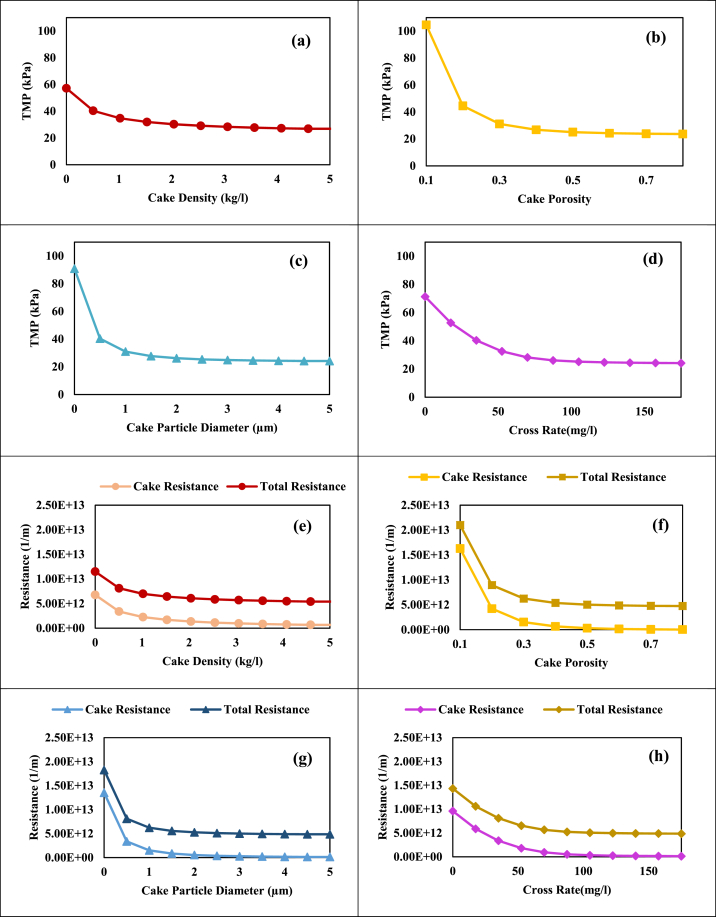
The sensitivity analysis results for the effects of: a, e) Cake density; b, f) Cake porosity; c, g) Cake particle diameter; d, h) Crossflow rate; on TMP, cake resistance, and total resistance, respectively.

Next, through sensitivity analysis (by method described in section 2, 3), the more accurate effect of cake density, cake porosity, cake particle diameter, and cross flow rate on the characteristics of the cake layer (in Fig. 5) and consequently on TMP and fouling (in Fig. 6) were investigated. Then suggestions were made to reduce membrane fouling in MBR.

Fig. 5-a and b reveal that increasing cake density or decreasing cake porosity leads to a thinner cake layer due to particle compaction, reduced particle spacing, and layer thickening. Conversely, a higher cross-flow rate diminishes cake thickness by generating shear stress on the membrane surface (Fig. 5-d). Variations in the cake layer's density (Fig. 5-a), porosity (Fig. 5-b), and particle diameter affect (Fig. 5-c) particle structure and distribution. However, the overall mass of the cake layer remains unchanged, as indicated in Fig. 5-e, f, and g, since the number and mass of particles remain constant. Higher cross-flow rates significantly decrease cake mass by cleaning the membrane surface and removing particles (Fig. 5-h). Therefore, it is advisable to reduce the cake layer's mass and thickness through shear stress creation on the membrane surface to improve membrane fouling.

Fig. 6-a illustrates that higher cake density lowers TMP by reducing particle spacing and cake layer thickness, which shortens the liquid flow path which leads to a significant drop in cake and total resistance (Fig. 6-e). Increased cake porosity and particle diameter, as shown in Fig. 6-b and c, create more open paths for flow, further reducing TMP which results in a sharp decrease in both cake and total resistance (Fig. 6-f and g). Fig. 6-d highlights that higher crossflow rates improve membrane cleaning and mass transfer, leading to thinner cake layers and lower TMP rate decreases both cake and total resistance (Fig. 6-h). Cake porosity has the greatest effect on reducing TMP and membrane fouling, as indicated in Fig. 6-b. To enhance cake layer porosity and reduce TMP, it is advisable to use anti-fouling materials (which control the formation of a cake layer on the surface of the membrane), regulate pH, and control temperature (by affecting the growth kinetics of microorganisms and preventing the growth of SMP and EPS), ultimately lowering operating costs.

There are various methods for controlling the fouling of membrane inside MBR along the operations. They include physical methods (such as aeration, backwashing, relaxation, vibration of the membrane module, ultrasound waves, back pulsing, and forward/back flushing), chemical methods (such as washing with acids, bases, and oxidizers), as well as a combination of them [91]. Indeed, the physical methods are mostly used for eliminating the fouling developed on the membrane surface, while the chemical methods are employed to resolve the fouling created inside the pores [92,93]. In addition, each of the mentioned methods has various controlling parameters. For example, the parameters affecting the aeration method include the size of air bubbles, intensity and duration of aeration [94]; the parameters influencing the backwashing method include the feed flow rate, pressure, temperature, duration, and the frequency of backwashing, concentration, pH, as well as the backwash solution compounds [95]; and the parameters affecting chemical methods included type, concentration, and pH of the chemical cleaner, temperature, pressure, as well as duration of contact with the chemical [91]. To achieve the best membrane performance along the filtration (minimum extent of fouling), optimization of the parameters of each method is essential. Table 10 presents the main strategy for reducing fouling before initiating the process as well as the strategy proposed for washing the membrane along the operations based on the mechanism of membrane fouling along with the values of the cake and pore resistances. According to the modeling results, the general strategy of washing (physical or chemical), and based on the simulation results, the intensity of washing operation (low, medium, and high) have been proposed.

According to Table 10, it is evident that in all studies, the cake resistance has been far larger than the pore resistance, suggesting that in MBR operational systems, due to the existence of sludge, the formation of the cake layer is very probable. This is also confirmed by the modeling results, in which all studies have matched the models based on cake formation, thus highlighting the necessity of adopting solutions for reducing fouling especially fouling resulting from cake layer along the operations. Since the research by Miyoshi, Yuasa [19] has matched the CF individual model, and the pore resistance is very low (0.392 × 10^+11^ 1/m), thus use of physical cleaning methods along the operations is sufficient for cleaning the membrane. However, the mechanism of the model of fouling in other studies presented in Table 10 has included both cake and pore fouling. Thus, to enhance the MBR efficiency, first physical methods are recommended for washing the cake layer, after which chemical methods could be used for resolving the resistance inside the pores. In the research by Zhao, Fu [75], the size of membrane pores has been smaller compared to other studies, leading to greater cake resistance [96], as also confirmed by the simulation results (according to Table 10). Since physical methods including aeration are recommended for resolving the cake fouling, and the cake resistance in this research has been far higher compared to other studies, physical washing with high intensity such as the use of coarse bubble aeration is recommended. Considering the process performed for washing in the noted research (usage of aeration with coarse bubbles instead of fine bubbles), again the accuracy and practicality of the suggestions resulting from the modeling and simulation results in reality are demonstrated. Thus, given the cake resistance values reported in Table 10, for the research by Zhao, Fu [75], Ibrahim, Sabeen [77], Han, Jia [78], Miyoshi, Yuasa [19], Yang, Chen [76], high-intensity physical washing methods; for studies by Wang, Li [4],Miyoshi, Yuasa [19], Erkan, Onkal Engin [58], Tay, Liu [68], Feng, Zhang [57], medium-intensity physical washing methods; and for the studies by Ouyang and Liu [20], Wang, Wu [73], Fallah, Bonakdarpour [79], Babatsouli, Palogos [85], low-intensity physical washing method are recommended. Considering the process adopted for membrane washing in the study by Tay, Liu [68], involving the usage of a medium-intensity physical method (first flushing with water within 2 min with 500 ml/min flow rate followed by backwashing with water within 15 min at 0.5 bar pressure), again the accuracy and practicality of the suggestions resulting from the modeling and simulation findings in actuality are proven. Meanwhile, for resolving pore fouling in the research by Zhao, Fu [75], Wang, Li [4], and Han, Jia [78], considering the larger pore resistance (according to Table 10), high-intensity chemical washing method; for studies by Ibrahim, Sabeen [77], Ouyang and Liu [20], Tay, Liu [68], Wang, Wu [73], Fallah, Bonakdarpour [79], Babatsouli, Palogos [85], and Yang, Chen [76], medium-intensity chemical washing method; and for studies by Miyoshi, Yuasa [19], Erkan, Onkal Engin [58], and Feng, Zhang [57], low-intensity chemical washing method are recommended. In the research by Yu, Yang [82], where the mechanism of fouling during the initial days is based on complete pore blocking, it is recommended that on the initial days of the process, only low-intensity physical washing methods be used. Nevertheless, since over time, the mechanism of development of fouling changes from complete pore blocking to complete pore blocking - standard pore blocking, in addition to physical cleaning, usage of chemical methods is essential for washing the membrane to minimize the extent of fouling. In addition, since the pore resistance is relatively high (2.29 × 10^+11^), medium-intensity chemical washing methods are suggested. Based on the process performed in this research, involved first flushing with water, followed by immersion in NaClO solution for 2 h at a concentration of 1000 mg/l, immersion in the citric acid solution for 2 h at a concentration of 1000 mg/l, and ultimately washing with water, the concordance of the mentioned suggestions with the actual operational process is observed. Therefore, it seems that by performing modeling and simulation together, the best solution for washing the membrane along the operations in MBR processes can be predicted for resolving the problem of membrane fouling, which is the most important challenge in the use of membrane processes.

## 4 Conclusion

This research aimed to identify the most efficient method for reducing membrane fouling in MBR systems through four steps:

1) Literature Review: It examined various MBR systems and identified pre-operation solutions to control fouling. Adjusting wastewater and biomass properties, such as increasing the C/N/P ratio from 5 to 10, tuning ionic strength, adding coagulants, and controlling pH, reduced TMP by 83 %, 85 %, 52 %, and 50 %, respectively. Enhancing membrane properties such as hydrophilicity and pore size reduced TMP by 75 % and 90 %, respectively. Operational adjustments, including temperature, SRT, HRT, and aeration, reduced fouling by 91 %, 85 %, 85 %, and 50 %, respectively.
2) Modeling: It selected the best model to describe fouling mechanisms and sites based on MSE and MAPE criteria. The results indicated accurate TMP predictions for various fouling models, with MSE values ranging from 0.02 to 3.8.
3) GPS-X Simulation: It simulated fouling quantities (cake and pore resistances, thickness, porosity, and mass) using a wide range of input parameters. The simulation accurately predicted MLSS and TMP variations over time, with MSE values up to 9.59 (g/l)^2^ and 6.91 (kPa)^2^. The accuracy of the simulations was confirmed by comparing predicted resistances with literature values.
4) Sensitivity Analysis: It identified cake layer porosity as the most effective factor in reducing membrane fouling. Increasing porosity from 10 % to 20 % lowered TMP from 105 to 45 kPa.

The key finding is that increasing cake layer porosity significantly reduces TMP and operating costs. To achieve optimal membrane performance, the study recommends physical and chemical cleaning methods based on modeling results, and suggests use of anti-fouling materials, pH regulation, and temperature control to enhance cake layer porosity.

## Data availability

Data will be made available on request.

## CRediT authorship contribution statement

**Maryam Homayoonfal:** Writing – review & editing, Supervision, Project administration, Methodology, Conceptualization. **Zohre Hajhashemi:** Writing – original draft, Validation, Investigation, Conceptualization. **Maryam Hajheidari:** Writing – original draft, Validation, Software, Investigation, Conceptualization. **Fateme Rezaei:** Writing – original draft, Software, Investigation, Data curation. **Mohammad Saber Nadali:** Software, Investigation.

## Declaration of competing interest

The authors declare that they have no known competing financial interests or personal relationships that could have appeared to influence the work reported in this paper.
