# Supplementary material for: Modeling and simulation-assisted strategies for effective membrane-fouling mitigation during membrane bioreactor operation
Source: Heliyon. 2024 Oct 4;10(20):e38953. doi: 10.1016/j.heliyon.2024.e38953 (PMC11531625; doi:10.1016/j.heliyon.2024.e38953)
Supplement: Multimedia component 1 [file mmc1.docx]

**Modeling and Simulation-Assisted Strategies for Effective Membrane-Fouling Mitigation during Membrane Bioreactor Operation**

**Maryam Homayoonfal^1,*^, Zohre Hajhashemi^1^, Maryam Hajheidari^1^, Fateme Rezaei^1^, Mohammad Saber Nadali^1^**

^1^ Department of Chemical Engineering, College of Engineering, University of Isfahan, P.O. Box 81746-73441, Isfahan, Iran

**^*^**^~^ corresponding author email address: [**m.homayoonfal@eng.ui.ac.ir**](mailto:m.homayoonfal@eng.ui.ac.ir)

**Table s-1. The amount of statistical indicators to evaluate the models for** [Wang, Li [1]](#_ENREF_1)

| **Model**  **Name** | **MSE**  **(*10^-6^)** | **RMSE (*10-^3^)** | **SSE**  **(*10^-8^)** | **MAE**  **(*10^-3^)** | **MAPE (*10^-1^)** | **MAX APE**  **(*10^-1^)** | **R^2^** | **SST**  **(*10^-8^)** |
| --- | --- | --- | --- | --- | --- | --- | --- | --- |
| **C** | 3.01 | 1.74 | 1.99 | 1.44 | 0.47 | 0.87 | 1 | 1.99 |
| **S** | 1.56 | 1.25 | 1.03 | 1.14 | 0.45 | 0.95 | 1 | 1.03 |
| **CF** | 33.96 | 5.83 | 22.41 | 5.26 | 2.15 | 4.53 | 1 | 22.41 |
| **I** | 9.58 | 3.1 | 6.32 | 2.86 | 1.13 | 2.04 | 1 | 6.32 |
| **CF-C** | FM | FM | FM | FM | FM | FM | FM | FM |
| **CF-I** | 7.09 | 2.66 | 4.68 | 2.47 | 0.97 | 1.77 | 1 | 4.68 |
| **C-S** | FM | FM | FM | FM | FM | FM | FM | FM |
| **I-S** | 1.56 | 1.25 | 1.03 | 1.14 | 0.45 | 0.95 | 1 | 1.03 |
| **CF-S** | 0.48 | 0.69 | 0.32 | 0.52 | 0.17 | 0.49 | 1 | 0.32 |

**Table s-2. The amount of statistical indicators to evaluate the models for** [Erkan, Onkal Engin [2]](#_ENREF_2)

| **Model**  **Name** | **MSE (*10^-6^)** | **RMSE (*10-^3^)** | **SSE (*10^-8^)** | **MAE (*10^-3^)** | **MAPE (*10^-1^)** | **MAX APE (*10^-1^)** | **R^2^** | **SST (*10^-8^)** |
| --- | --- | --- | --- | --- | --- | --- | --- | --- |
| **C** | 260.00 | 16.10 | 159.00 | 13.30 | 10.10 | 17.50 | 1 | 159.00 |
| **S** | 26.70 | 5.17 | 16.30 | 4.54 | 4.37 | 6.52 | 1 | 16.30 |
| **CF** | 21.00 | 4.58 | 12.80 | 3.90 | 4.49 | 11.30 | 1 | 12.80 |
| **I** | 0.46 | 0.68 | 0.28 | 0.58 | 1.21 | 5.03 | 1 | 0.28 |
| **CF-C** | 0.43 | 0.65 | 0.26 | 0.55 | 0.88 | 4.30 | 1 | 0.26 |
| **CF-I** | 0.46 | 0.68 | 0.28 | 0.58 | 1.21 | 5.03 | 1 | 0.28 |
| **C-S** | 26.70 | 5.17 | 16.30 | 4.54 | 4.37 | 6.52 | 1 | 16.30 |
| **I-S** | 0.46 | 0.68 | 0.28 | 0.58 | 1.21 | 5.03 | 1 | 0.28 |
| **CF-S** | 1.90 | 1.38 | 1.16 | 1.15 | 1.28 | 3.26 | 1 | 1.16 |

**Table s-3. The amount of statistical indicators to evaluate the models for** [Ibrahim, Sabeen [3]](#_ENREF_3)

| **Model**  **Name** | **MSE (*10^-6^)** | **RMSE**  **(*10-^3^)** | **SSE**  **(*10^-8^)** | **MAE**  **(*10^-3^)** | **MAPE**  **(*10^-1^)** | **MAX APE**  **(*10^-1^)** | **R^2^** | **SST**  **(*10^-8^)** |
| --- | --- | --- | --- | --- | --- | --- | --- | --- |
| **C** | 404.29 | 20.11 | 270.87 | 16.42 | 10.54 | 25.99 | 1 | 270.87 |
| **S** | 11.08 | 3.33 | 7.43 | 2.88 | 3.08 | 5.91 | 1 | 7.43 |
| **CF** | 35.29 | 5.94 | 23.64 | 4.75 | 3.33 | 7.24 | 1 | 23.64 |
| **I** | 6.31 | 2.51 | 4.23 | 2.13 | 1.92 | 5 | 1 | 4.23 |
| **CF-C** | 3.48 | 1.87 | 2.33 | 1.58 | 1.75 | 4.92 | 1 | 2.33 |
| **CF-I** | 4.97 | 2.23 | 3.33 | 1.90 | 1.94 | 5.15 | 1 | 3.33 |
| **C-S** | FM | FM | FM | FM | FM | FM | FM | FM |
| **I-S** | 4.25 | 2.06 | 2.85 | 1.74 | 1.96 | 5.20 | 1 | 2.85 |
| **CF-S** | 1.66 | 1.29 | 1.11 | 1.05 | 1.12 | 3.95 | 1 | 1.11 |

**Table s-4. The amount of statistical indicators to evaluate the models for** [Ouyang and Liu [4]](#_ENREF_4)

| **Model**  **Name** | **MSE**  **(*10^-5^)** | **RMSE**  **(*10^-2^)** | **SSE**  **(*10^-7^)** | **MAE**  **(*10^-2^)** | **MAPE**  **(*10^-1^)** | **MAX APE**  **(*10^-1^)** | **R^2^** | **SST**  **(*10^-7^)** |
| --- | --- | --- | --- | --- | --- | --- | --- | --- |
| **C** | 3.61 | 6.01 | 1.41 | 4.36 | 1.44 | 4.52 | 1 | 1.41 |
| **S** | 1.96 | 4.43 | 0.76 | 3.99 | 1.74 | 5.31 | 1 | 0.76 |
| **CF** | 9.08 | 9.53 | 3.54 | 9.04 | 3.85 | 9.44 | 1 | 3.54 |
| **I** | 3.31 | 5.75 | 1.29 | 5.36 | 2.46 | 6.64 | 1 | 1.29 |
| **CF-C** | 2.04 | 4.52 | 0.80 | 4.04 | 1.73 | 5.31 | 1 | 0.80 |
| **CF-I** | 2.78 | 5.28 | 1.09 | 4.91 | 2.27 | 6.29 | 1 | 1.09 |
| **C-S** | 1.95 | 4.41 | 0.76 | 3.94 | 1.69 | 5.22 | 1 | 0.76 |
| **I-S** | 1.96 | 4.43 | 0.76 | 3.99 | 1.74 | 5.31 | 1 | 0.76 |
| **CF-S** | 1.83 | 4.28 | 0.71 | 3.59 | 1.45 | 4.65 | 1 | 0.71 |

**Table s-5. The amount of statistical indicators to evaluate the models for** [Feng, Zhang [5]](#_ENREF_5)

| **Model**  **Name** | **MSE (*10^-6^)** | **RMSE**  **(*10-^3^)** | **SSE**  **(*10^-8^)** | **MAE**  **(*10^-3^)** | **MAPE**  **(*10^-1^)** | **MAX APE**  **(*10^-1^)** | **R^2^** | **SST**  **(*10^-8^)** |
| --- | --- | --- | --- | --- | --- | --- | --- | --- |
| **C** | 1.48 | 1.22 | 1.84 | 0.96 | 0.89 | 2.59 | 1 | 1.84 |
| **S** | 2 | 1.41 | 2.48 | 1.18 | 1.38 | 3.52 | 1 | 2.48 |
| **CF** | 22.83 | 4.78 | 28.31 | 3.97 | 4.01 | 9.26 | 1 | 28.31 |
| **I** | 8.51 | 2.92 | 10.55 | 2.43 | 2.53 | 5.71 | 1 | 10.55 |
| **CF-C** | FM | FM | FM | FM | FM | FM | FM | FM |
| **CF-I** | 6.67 | 2.58 | 8.27 | 2.16 | 2.28 | 5.28 | 1 | 8.27 |
| **C-S** | FM | FM | FM | FM | FM | FM | FM | FM |
| **I-S** | 2 | 1.41 | 2.48 | 1.18 | 1.38 | 3.52 | 1 | 2.48 |
| **CF-S** | 0.5 | 0.71 | 0.62 | 0.54 | 0.76 | 3.08 | 1 | 0.62 |

**Table s-6. The amount of statistical indicators to evaluate the models for** [Wang, Wu [6]](#_ENREF_6)

| **Model**  **Name** | **MSE**  **(*10^-6^)** | **RMSE**  **(*10^-3^)** | **SSE (*10^-7^)** | **MAE**  **(*10^-2^)** | **MAPE** | **MAX APE**  **(*10^-1^)** | **R^2^** | **SST (*10^-7^)** |
| --- | --- | --- | --- | --- | --- | --- | --- | --- |
| **C** | 1.74 | 1.32 | 5.90 | 8.66 | 7.59 | 2.54 | 1 | 5.90 |
| **S** | 1.58 | 1.26 | 5.38 | 9.69 | 9.80 | 2.29 | 1 | 5.38 |
| **CF** | 8.17 | 2.86 | 27.80 | 25.00 | 28.00 | 6.50 | 1 | 27.80 |
| **I** | 3.31 | 1.82 | 11.20 | 16.30 | 17.50 | 3.62 | 1 | 11.20 |
| **CF-C** | 1.35 | 1.16 | 4.60 | 7.36 | 6.81 | 2.13 | 1 | 4.60 |
| **CF-I** | 2.78 | 1.67 | 9.44 | 14.70 | 15.70 | 3.27 | 1 | 9.44 |
| **C-S** | 1.35 | 1.16 | 4.59 | 7.32 | 6.74 | 2.12 | 1 | 4.59 |
| **I-S** | 1.58 | 1.26 | 5.38 | 9.69 | 9.80 | 2.29 | 1 | 5.38 |
| **CF-S** | 1.32 | 1.15 | 4.50 | 7.02 | 6.06 | 2.06 | 1 | 4.50 |

**Table s-7. The amount of statistical indicators to evaluate the models for** [Fallah, Bonakdarpour [7]](#_ENREF_7)

| **Model**  **Name** | **MSE**  **(*10^-5^)** | **RMSE**  **(*10^-2^)** | **SSE**  **(*10^-6^)** | **MAE**  **(*10^-2^)** | **MAPE**  **(*10^-1^)** | **MAX APE**  **(*10^-1^)** | **R^2^** | **SST**  **(*10^-6^)** |
| --- | --- | --- | --- | --- | --- | --- | --- | --- |
| **C** | 1.78 | 4.21 | 6.57 | 3.54 | 2.14 | 4.46 | 1 | 6.57 |
| **S** | 1.52 | 3.90 | 5.62 | 3.20 | 1.94 | 4.18 | 1 | 5.62 |
| **CF** | 0.96 | 3.09 | 3.54 | 2.48 | 1.56 | 3.30 | 1 | 3.54 |
| **I** | 1.29 | 3.59 | 4.78 | 2.92 | 1.79 | 3.87 | 1 | 4.78 |
| **CF-C** | 0.63 | 2.50 | 2.32 | 2.14 | 1.48 | 5.26 | 1 | 2.32 |
| **CF-I** | 0.61 | 2.47 | 2.25 | 2.07 | 1.34 | 3.17 | 1 | 2.25 |
| **C-S** | 1.52 | 3.90 | 5.62 | 3.20 | 1.94 | 4.18 | 1 | 5.62 |
| **I-S** | 0.57 | 2.39 | 2.11 | 1.99 | 1.33 | 3.93 | 1 | 2.11 |
| **CF-S** | 0.96 | 3.09 | 3.54 | 2.48 | 1.56 | 3.30 | 1 | 3.54 |

**Table s-8. The amount of statistical indicators to evaluate the models for** [Han, Jia [8]](#_ENREF_8)

| **Model**  **Name** | **MSE**  **(*10^-6^)** | **RMSE**  **(*10-^3^)** | **SSE**  **(*10^-8^)** | **MAE**  **(*10^-3^)** | **MAPE**  **(*10^-1^)** | **MAX APE**  **(*10^-1^)** | **R^2^** | **SST**  **(*10^-8^)** |
| --- | --- | --- | --- | --- | --- | --- | --- | --- |
| **C** | 470.82 | 21.7 | 409.61 | 18.76 | 10.0 | 10.0 | 1 | 470.82 |
| **S** | 96.167 | 9.81 | 83.67 | 8.35 | 4.52 | 6.60 | 1 | 96.17 |
| **CF** | 8.08 | 2.84 | 7.03 | 2.47 | 3.39 | 14.77 | 1 | 8.08 |
| **I** | 26.82 | 5.18 | 23.34 | 4.39 | 2.44 | 4.01 | 1 | 26.82 |
| **CF-C** | 2.79 | 1.7 | 2.43 | 1.46 | 1.98 | 9.61 | 1 | 2.79 |
| **CF-I** | 2.56 | 1.6 | 2.23 | 1.4 | 1.87 | 9.09 | 1 | 2.56 |
| **C-S** | FM | FM | FM | FM | FM | FM | FM | FM |
| **I-S** | 1.26 | 1.12 | 1.1 | 0.96 | 0.78 | 3.28 | 1 | 1.26 |
| **CF-S** | 4.3 | 2.07 | 3.74 | 1.8 | 2.52 | 11.86 | 1 | 4.3 |

**Table s-9. The amount of statistical indicators to evaluate the models for** [Yu, Yang [9]](#_ENREF_9)**-part 1**

| **Model**  **Name** | **MSE**  **(*10^-5^)** | **RMSE**  **(*10^-2^)** | **SSE**  **(*10^-6^)** | **MAE**  **(*10^-2^)** | **MAPE**  **(*10^-1^)** | **MAX APE**  **(*10^-1^)** | **R^2^** | **SST**  **(*10^-6^)** |
| --- | --- | --- | --- | --- | --- | --- | --- | --- |
| **C** | 1.42 | 3.76 | 4.39 | 3.20 | 1.09 | 3.71 | 1 | 4.39 |
| **S** | 1.97 | 4.43 | 6.09 | 3.62 | 1.20 | 4.11 | 1 | 6.09 |
| **CF** | 8.46 | 9.20 | 26.20 | 7.67 | 2.36 | 6.24 | 1 | 26.20 |
| **I** | 3.85 | 6.20 | 11.90 | 4.87 | 1.48 | 4.64 | 1 | 11.90 |
| **CF-C** | 1.34 | 3.66 | 4.16 | 3.27 | 1.12 | 3.80 | 1 | 4.16 |
| **CF-I** | 3.32 | 5.77 | 10.30 | 4.48 | 1.38 | 4.49 | 1 | 10.30 |
| **C-S** | 1.35 | 3.67 | 4.17 | 3.27 | 1.12 | 3.79 | 1 | 4.17 |
| **I-S** | 1.97 | 4.43 | 6.09 | 3.63 | 1.20 | 4.11 | 1 | 6.09 |
| **CF-S** | 1.61 | 4.01 | 4.99 | 3.56 | 1.18 | 3.55 | 1 | 4.99 |

**Table s-10. The amount of statistical indicators to evaluate the models for** [Yu, Yang [9]](#_ENREF_9)**-part 2**

| **Model**  **Name** | **MSE**  **(*10^-7^)** | **RMSE**  **(*10^-3^)** | **SSE**  **(*10^-8^)** | **MAE**  **(*10^-3^)** | **MAPE**  **(*10^-1^)** | **MAX APE**  **(*10^-1^)** | **R^2^** | **SST**  **(*10^-8^)** |
| --- | --- | --- | --- | --- | --- | --- | --- | --- |
| **C** | 1.88 | 4.33 | 9.39 | 3.05 | 2.42 | 5.56 | 1 | 9.39 |
| **S** | 1.96 | 4.42 | 9.78 | 3.03 | 2.38 | 5.73 | 1 | 9.78 |
| **CF** | 2.11 | 4.60 | 10.60 | 3.00 | 2.30 | 6.03 | 1 | 10.60 |
| **I** | 2.02 | 4.49 | 10.10 | 3.02 | 2.35 | 5.85 | 1 | 10.10 |
| **CF-C** | 1.83 | 4.28 | 9.17 | 3.07 | 2.45 | 5.46 | 1 | 9.17 |
| **CF-I** | 2.00 | 4.48 | 10.00 | 3.02 | 2.35 | 5.83 | 1 | 10.00 |
| **C-S** | 0.13 | 1.12 | 0.63 | 1.00 | 0.89 | 2.01 | 1 | 0.63 |
| **I-S** | 1.96 | 4.42 | 9.78 | 3.03 | 2.38 | 5.73 | 1 | 9.78 |
| **CF-S** | 0.98 | 3.14 | 4.92 | 2.15 | 1.67 | 4.23 | 1 | 4.92 |

**Table s-11. The amount of statistical indicators to evaluate the models for** [Zhao, Fu [10]](#_ENREF_10)

| **Model**  **Name** | **MSE**  **(*10^-6^)** | **RMSE**  **(*10^-3^)** | **SSE**  **(*10^-8^)** | **MAE**  **(*10^-3^)** | **MAPE**  **(*10^-1^)** | **MAX APE**  **(*10^-1^)** | **R^2^** | **SST**  **(*10^-7^)** |
| --- | --- | --- | --- | --- | --- | --- | --- | --- |
| **C** | 260.00 | 16.10 | 159.00 | 13.30 | 10.10 | 17.50 | 1 | 1590.0 |
| **S** | 26.70 | 5.17 | 16.30 | 4.54 | 4.37 | 6.52 | 1 | 163.0 |
| **CF** | 21.00 | 4.58 | 12.80 | 3.90 | 4.49 | 11.30 | 1 | 128.0 |
| **I** | 0.46 | 0.68 | 0.28 | 0.58 | 1.21 | 5.03 | 1 | 2.80 |
| **CF-C** | 0.43 | 0.65 | 0.26 | 0.55 | 0.88 | 4.30 | 1 | 2.60 |
| **CF-I** | 0.46 | 0.68 | 0.28 | 0.58 | 1.21 | 5.03 | 1 | 2.79 |
| **C-S** | 26.70 | 5.17 | 16.30 | 4.54 | 4.37 | 6.52 | 1 | 163.0 |
| **I-S** | 0.46 | 0.68 | 0.28 | 0.58 | 1.21 | 5.03 | 1 | 2.79 |
| **CF-S** | 1.90 | 1.38 | 1.16 | 1.15 | 1.28 | 3.26 | 1 | 11.60 |

**Table s-12. The amount of statistical indicators to evaluate the models for** [Tay, Liu [11]](#_ENREF_11)

| **Model**  **Name** | **MSE**  **(*10^-6^)** | **RMSE**  **(*10^-3^)** | **SSE**  **(*10^-8^)** | **MAE**  **(*10^-3^)** | **MAPE**  **(*10^-1^)** | **MAX APE**  **(*10^-1^)** | **R^2^** | **SST**  **(*10^-8^)** |
| --- | --- | --- | --- | --- | --- | --- | --- | --- |
| **C** | 367.0 | 19.20 | 198.0 | 17.90 | 11.0 | 21.10 | 1 | 198.00 |
| **S** | 6.15 | 2.48 | 3.32 | 1.89 | 1.14 | 3.31 | 1 | 3.32 |
| **CF** | 14.20 | 3.77 | 7.67 | 2.72 | 1.63 | 5.07 | 1 | 7.67 |
| **I** | 8.74 | 2.96 | 4.72 | 2.20 | 1.32 | 3.71 | 1 | 4.72 |
| **CF-C** | 3.83 | 1.96 | 2.07 | 1.56 | 1.0 | 2.73 | 1 | 2.07 |
| **CF-I** | 8.07 | 2.84 | 4.36 | 2.13 | 1.27 | 3.48 | 1 | 4.36 |
| **C-S** | 3.82 | 1.95 | 2.06 | 1.56 | 1.02 | 2.82 | 1 | 2.06 |
| **I-S** | 6.15 | 2.48 | 3.32 | 1.89 | 1.14 | 3.30 | 1 | 3.32 |
| **CF-S** | 5.08 | 2.25 | 2.74 | 1.81 | 1.15 | 2.97 | 1 | 2.74 |

**Table s-13. The amount of statistical indicators to evaluate the models for** [Miyoshi, Yuasa [12]](#_ENREF_12)

| **Model**  **Name** | **MSE**  **(*10^-7^)** | **RMSE**  **(*10^-4^)** | **SSE**  **(*10^-9^)** | **MAE**  **(*10^-4^)** | **MAPE**  **(*10^-1^)** | **MAX APE**  **(*10^-1^)** | **R^2^** | **SST**  **(*10^-9^)** |
| --- | --- | --- | --- | --- | --- | --- | --- | --- |
| **C** | 27.20 | 1.65 | 17.10 | 1.44 | 9.41 | 12.30 | -2.9 | -5.91 |
| **S** | 18.90 | 1.38 | 11.90 | 1.24 | 8.62 | 9.66 | 1 | 12.0 |
| **CF** | 0.38 | 0.19 | 0.24 | 0.17 | 1.98 | 9.06 | 1 | 0.24 |
| **I** | 7.63 | 0.87 | 4.80 | 0.80 | 6.59 | 9.49 | 1 | 4.81 |
| **CF-C** | 24.40 | 1.56 | 15.40 | 1.36 | 9.13 | 9.75 | 1 | 15.50 |
| **CF-I** | 4.48 | 0.67 | 2.82 | 0.60 | 5.60 | 9.48 | 1 | 2.83 |
| **C-S** | 18.90 | 1.38 | 11.90 | 1.24 | 8.62 | 9.66 | 1 | 12.0 |
| **I-S** | 3.09 | 0.56 | 1.95 | 0.49 | 4.92 | 9.45 | 0.55 | 3.54 |
| **CF-S** | 0.38 | 0.20 | 0.24 | 0.17 | 1.98 | 9.06 | 1 | 0.24 |

**Table s-14. The amount of statistical indicators to evaluate the models for** [Babatsouli, Palogos [13]](#_ENREF_13)

| **Model**  **Name** | **MSE**  **(*10^-6^)** | **RMSE (*10-^3^)** | **SSE**  **(*10^-8^)** | **MAE**  **(*10^-3^)** | **MAPE (*10^-1^)** | **MAX APE**  **(*10^-1^)** | **R^2^** | **SST**  **(*10^-8^)** |
| --- | --- | --- | --- | --- | --- | --- | --- | --- |
| **C** | 181.21 | 13.47 | 38.06 | 12.54 | 12.7 | 34.45 | 1 | 38.06 |
| **S** | 12.94 | 3.6 | 2.72 | 2.12 | 1.56 | 4.77 | 1 | 2.72 |
| **CF** | 29.81 | 5.46 | 6.26 | 4.67 | 4.58 | 8.34 | 1 | 6.26 |
| **I** | 6.38 | 2.53 | 1.34 | 1.78 | 1.44 | 3.07 | 1 | 1.34 |
| **CF-C** | 10.83 | 3.3 | 2.28 | 2.76 | 2.49 | 4.8 | 1 | 2.28 |
| **CF-I** | 9.95 | 3.16 | 2.09 | 2.62 | 2.34 | 4.49 | 1 | 2.09 |
| **C-S** | FM | FM | FM | FM | FM | FM | FM | FM |
| **I-S** | 138.4 | 11.77 | 29.07 | 10.34 | 9.07 | 10 | 1 | 29.07 |
| **CF-S** | 12.68 | 3.57 | 2.67 | 3.05 | 2.81 | 5.38 | 1 | 2.67 |

**Table s-15. The amount of statistical indicators to evaluate the models for** [Yang, Chen [14]](#_ENREF_14)

| **Model**  **Name** | **MSE**  **(*10^-6^)** | **RMSE (*10-^3^)** | **SSE**  **(*10^-8^)** | **MAE**  **(*10^-3^)** | **MAPE (*10^-1^)** | **MAX APE**  **(*10^-1^)** | **R^2^** | **SST**  **(*10^-8^)** |
| --- | --- | --- | --- | --- | --- | --- | --- | --- |
| **C** | 25.4 | 5.04 | 13.72 | 4.52 | 2.68 | 4.05 | 1 | 13.72 |
| **S** | 8.75 | 2.96 | 4.73 | 2.63 | 1.59 | 2.58 | 1 | 4.73 |
| **CF** | 4.35 | 2.09 | 2.35 | 1.85 | 1.46 | 5.57 | 1 | 2.35 |
| **I** | 1.12 | 1.06 | 0.61 | 0.91 | 0.55 | 2.55 | 1 | 0.61 |
| **CF-C** | 0.42 | 0.65 | 0.23 | 0.57 | 0.48 | 3.46 | 1 | 0.23 |
| **CF-I** | 0.22 | 0.47 | 0.12 | 0.39 | 0.31 | 2.87 | 1 | 0.12 |
| **C-S** | FM | FM | FM | FM | FM | FM | FM | FM |
| **I-S** | 0.23 | 0.48 | 0.13 | 0.4 | 0.33 | 2.93 | 1 | 0.13 |
| **CF-S** | 0.62 | 0.79 | 0.34 | 0.69 | 0.59 | 3.87 | 1 | 0.34 |

**References**

1. Wang, Y., J. Li, and J. Zhu, *Comparative analysis of membrane fouling mechanisms induced by operation modes of membrane bioreactors with aerobic granular sludge.* Heliyon, 2023. **9**(7): p. e17973.

2. Erkan, H.S., et al., *Effect of carbon to nitrogen ratio of feed wastewater and sludge retention time on activated sludge in a submerged membrane bioreactor.* Environ Sci Pollut Res Int, 2016. **23**(11): p. 10742-10752.

3. Ibrahim, R.S., et al., *Comparative study of suspended and attached growth in membrane bioreactors for wastewater treatment.* Water and Environment Journal, 2020. **34**(S1): p. 273-289.

4. Ouyang, K. and J. Liu, *Effect of sludge retention time on sludge characteristics and membrane fouling of membrane bioreactor.* Journal of Environmental Sciences, 2009. **21**(10): p. 1329-1335.

5. Feng, S., et al., *The effect of COD/N ratio on process performance and membrane fouling in a submerged bioreactor.* Desalination, 2012. **285**: p. 232-238.

6. Wang, Z., Z. Wu, and S. Tang, *Impact of Temperature Seasonal Change on Sludge Characteristics and Membrane Fouling in a Submerged Membrane Bioreactor.* Separation Science and Technology, 2010. **45**(7): p. 920-927.

7. Fallah, N., et al., *Long-term operation of submerged membrane bioreactor (MBR) for the treatment of synthetic wastewater containing styrene as volatile organic compound (VOC): Effect of hydraulic retention time (HRT).* J Hazard Mater, 2010. **178**(1-3): p. 718-24.

8. Han, J., et al., *Effect of magnetic field coupled magnetic biochar on membrane bioreactor efficiency, membrane fouling mitigation and microbial communities.* Science of The Total Environment, 2024. **931**: p. 172549.

9. Yu, L., et al., *Effects of solids retention time on the performance and microbial community structures in membrane bioreactors treating synthetic oil refinery wastewater.* Chemical Engineering Journal, 2018. **344**: p. 462-468.

10. Zhao, D., et al., *Effects of coarse and fine bubble aeration on performances of membrane filtration and denitrification in moving bed membrane bioreactors.* Science of The Total Environment, 2021. **772**: p. 145513.

11. Tay, M.F., et al., *The feasibility of nanofiltration membrane bioreactor (NF-MBR)+ reverse osmosis (RO) process for water reclamation: Comparison with ultrafiltration membrane bioreactor (UF-MBR)+ RO process.* Water research, 2018. **129**: p. 180-189.

12. Miyoshi, T., et al., *Effect of membrane polymeric materials on relationship between surface pore size and membrane fouling in membrane bioreactors.* Applied Surface Science, 2015. **330**: p. 351-357.

13. Babatsouli, P., et al., *Evaluation of a MBR Pilot Treating Industrial Wastewater with a High COD/N Ratio.* Journal of Chemical Technology and Biotechnology, 2015. **90**.

14. Yang, Q., J. Chen, and F. Zhang, *Membrane fouling control in a submerged membrane bioreactor with porous, flexible suspended carriers.* Desalination, 2006. **189**(1): p. 292-302.
